# Situation Change: Stability and Change of Situation Variables between and within Persons

**DOI:** 10.3389/fpsyg.2015.01938

**Published:** 2016-01-06

**Authors:** John F. Rauthmann, Ryne A. Sherman

**Affiliations:** ^1^Department of Psychology, Personality Psychology, Humboldt-Universität zu BerlinBerlin, Germany; ^2^Department of Psychology, Florida Atlantic UniversityBoca Raton, FL, USA

**Keywords:** situations, situation change, person-situation transactions, situation management strategies, Situational Eight DIAMONDS, individual differences

## Abstract

When, how, and why situations flow into one another is important for understanding dynamic personality processes, but the topic of situation change has traditionally been a thorny issue in personality/social psychology. We explore conceptual and methodological issues in research on situation change: (1) What is situation change, which variables could we measure, and how can situation change be methodologically captured and analyzed (at between- and within-person levels)? (2) Which person-situation transaction mechanisms (situation management strategies) could entail stability and change of situations in daily life? (3) How do single or repeated instances of situation change impact short-, middle-, and long-term outcomes (e.g., intra- and interpersonal adjustment)? Besides laying out a research program for situation change, we present preliminary data from participants who wore mini-video cameras recording their situations so that they could be rated later in the lab. We demonstrate rater consensus on when situations change, mean-level changes of situation characteristics across situations, similarity of situation characteristics across adjacent situations, and inter-individual differences in intra-individual situation change in change networks.

## Situation change

**Figure 1:**
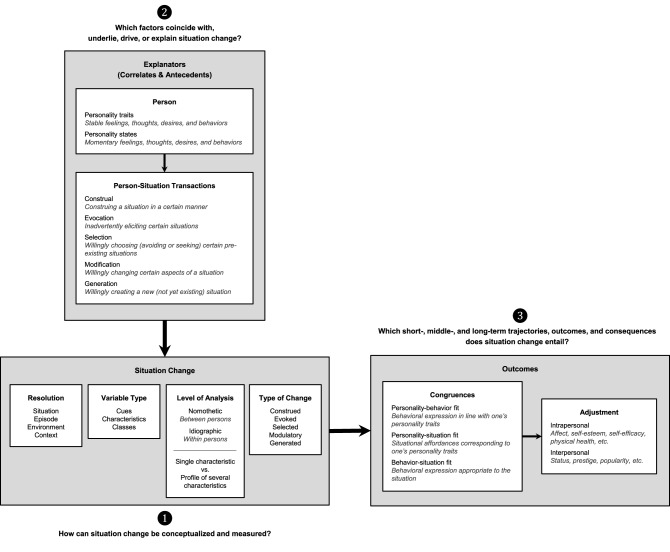
**A research program for situation change**.

When, how, and why does one situation end and another other begin? Studying *situation change* has been a thorny issue in psychology for several reasons. Research on situations in general has faced recurring problems, such as the conceptualization, taxonomization, and measurement of situational information (Hogan, 2009). The lack of a clear and consensual understanding of what situations are and how they can be described and measured obviously makes the study of situation change practically impossible. Thus, the topic of situation change—as the stability vs. variability of situations or how situations flow into each other—has received relatively little attention so far although its importance has been already understood (e.g., Argyle et al., 1981; Magnusson, 1981c). Recently, however, situation research has begun to receive renewed interest and increasing attention (Reis, 2008), resulting in several advances that may be useful when studying situation change (e.g., Rauthmann et al., 2015a,b). As such, this article seeks to lay the foundation for such research by addressing three major questions (along with specific sub-questions; see Figure 1):

1. Conceptualization and Measurement:
  What is situation change? How can it be captured and studied?
2. Correlates and Antecedents:
  *Which variables explain (* = *coincide with or predict) situation change?*
3. Trajectories and Outcomes:
  How does situation change unfold? Which variables does situation change predict?

## The importance of (studying) situation change

Before we address the three major issues outlined above, we summarize reasons why it is important to study situation change in the first place. First, most of psychology (save developmental psychology) is focused rather on static structures. As such, much of situation research is devoted to understanding “the situation” or certain (experimentally manipulated) stimuli. In such research, situational variables are static in the sense that they do not or cannot change. However, real life is lived in a flowing stream of situations that are ever changing. If it is our goal to understand the everyday lives of people, we must develop theories and methods to study dynamic aspects of situations. Elaborations on situation change should serve to fill this lacuna. Second, while it is important to acknowledge that situations change at all, it will be good to know *how* (i.e., in which ways) they change. The types of situation change may tell us something about the people in those changed or changing situations. If it is our goal to predict behavior (not just central tendencies such as the mean, but also other parameters of entire density distributions of personality expressions; see Fleeson and Gallagher, 2009), we should also take into account in which ways the situations change. For example, some situations may change suddenly and abruptly, while others may drag along and change gradually. In such cases, different behavioral processes will undoubtedly be involved. Third, understanding *why* situations change will elucidate person-situation transactions, or more specifically, “person-to-situation” transactions. Personality and situation characteristics are correlated (Ickes et al., 1997; Rauthmann et al., 2015c), and these correlations may emerge because of what people “do” to their situations (and also what these situations, cumulatively over time, do to them). How people navigate and “manage” their daily situations should, to a great deal, determine further information processing, behavior, and other outcomes (e.g., health). For example, in the corresponsive principle of personality development (Roberts and Wood, 2006), it is posited that people seek out situations that “fit” their personalities, while those sought after situations, in turn, deepen and consolidate the personalities that have led to seeking them out. Such person-situation transactions in personality development could benefit from better understanding situation change.

## Conceptualizing and measuring situation change

The measurement of situation change hinges upon how it is defined. Generally, three broad questions need to be answered:

1. Resolution: *At what level of abstraction are “situational variables” located?*
2. Variable Type: *Which types of “situational variables” are used?*
3. Analytic Level: *Are analyses conducted nomothetically (between-person level) or idiographically (within-person level)?*

### Resolution

Rauthmann et al. (2015b) clarified that there are different phenomena that have been referred to as “the situation” in extant theory and research: situation, episode, environment, and context. These terms are hierarchically nested within each other. Several situations (e.g., being greeted by friends, getting something to drink, listening to loud music, a vivid conversation, etc.) can be linked together so that they form an ongoing episode (e.g., a party episode with many different situations in it). Situations and episodes are embedded into the environment of a person (i.e., one's habitual socio-ecological surroundings) which itself is, in turn, couched into a larger context (e.g., history, epoch, zeitgeist, socio-culture). This work is concerned with situations as momentary and fleeting phenomena that dynamically flow into each other. It is the flow, or the segmentation of this flow, that is so daunting to situation change research. In examining stability and change of situations, we inevitably will also touch upon episodes which are at a lower resolution because they are more abstract (and could potentially subsume several situations that have changed yet are still sufficiently similar to group together). Though the change of one's environment (and context) is also an interesting topic, this presupposes that we have knowledge on situation change because environments are, to a great part, a function of recurring, typical, or “crystallized” situations and episodes of a person (Rauthmann et al., 2015b).

### Variable type

#### Objective vs. subjective demarcations

Demarcations of situations can be generally viewed from a more objective or more subjective perspective (Fiske, 1977; Raush, 1977; Craik, 1981; Magnusson, 1981a,b,c). The objective perspective stresses either (a) physically existent or “objectively” quantifiable information in the environment (stimuli) or (b) consensually agreed upon “quasi-objective” facts, while the subjective perspective, in contrast, experiences or perceptions of people (that need not be shared with others, but can be idiosyncratic; see Rauthmann, 2012 and Rauthmann et al., 2015a,b for details).

This basic distinction is important to the question of situation change. For example, the episode “going home from the gym” includes (at least) three spatially distinct environments: gym, way home, at home. This could imply three physically demarcated situations, yet the psychological situation of the walking individual may not have changed within these three environmental settings (Stebbins, 1969) as he/she might have been thinking all the walking time about what to cook later (and would thus classify the entire situation episode as “planning what to do”). So: Has the “situation” changed or not? In objective terms it has (because of the variation in the physical world), but in subjective terms it has not (because of no variation in the cognitively represented world). However, there are also examples, where a change in space results in a change in the (perceived) situation as different rules and roles become salient and predominant. Suppose the individual from before comes home, greets his/her spouse (room: hallway; role: spouse), goes on to play with the kids (room: children's room; role: parent), then cooks dinner (room: kitchen; role: chef), eats with family (room: dining room; roles: spouse + parent), and after that works on a project for the meeting the next day before going to bed (room: home office; role: worker). In this example, the different rooms are associated with different roles which are, in turn, associated with different generative behavioral rules (Argyle et al., 1981). Thus, situations may be demarcated in physical *and* psychological terms as each room comes attached with different meanings, roles, and rules. Taken together, there may be discontinuities in the physical and psychological situations with various transitions to different situational structures (Magnusson, 1981a,b,c).

#### Situational information

Generally, there are three types of situational information (Rauthmann, 2015; Rauthmann et al., 2015c): cues, characteristics, and classes. *Cues* (e.g., amount of people, number of books, lighting, noise, etc.) circumscribe distal stimuli in the physical environment that are objectively measurable. They have been mainly used for experimental social-psychological research and often comprise PEARLS (Noftle and Gust, 2015): *p*ersons (any other persons around someone), *e*vents (anything happening around someone), *a*ctivities (what people are doing), *r*oles (the formal and social roles that people inhabit), *l*ocation (the space and time in which a situation is couched), and *s*tates (people's ambient thoughts, feelings, and desires). Note here that particularly roles and states pertain more to aspects of or within a person that accompany a situation, and do not necessarily belong to or define it (Rauthmann et al., 2015a).

*Characteristics* (e.g., intellectual, adverse, terrifying, etc.) describe meanings and interpretations that people form about single or multiple cues once they have explicitly and/or implicitly processed them. They can be used to describe situations similar to how people are described with personality dimensions (de Raad, 2004; Edwards and Templeton, 2005). Recently, Rauthmann et al. (2014) proposed to taxonomize situation characteristics into the Situational Eight DIAMONDS Model, containing *D*uty (Does work need to be done?), *I*ntellect (Is deep cognitive information processing relevant?), *A*dversity (Is someone under threat?), *M*ating (Is the situation erotically charged?), p*O*sitivity (Is the situation enjoyable?), *N*egativity (Could the situation turn negative?), *D*eception (Is mistrust an issue?), and *S*ociality (Is meaningful social interaction and relationship building possible?). This taxonomy integrates most dimensions from previous situation literature (see Rauthmann, 2015 for a review) and also includes some that have not been routinely found (i.e., Intellect, Deception). Additionally, it has already spawned well-validated assessment tools (32-item measure: Rauthmann et al., 2014; 24-item measure: Rauthmann and Sherman, 2015a; 8-item ultra-short measure: Rauthmann and Sherman, 2015b). Further, the DIAMONDS model has proven useful in substantive empirical research, including (a) predicting personality expressions in an experience sampling study (Sherman et al., 2015), (b) understanding the temporal contiguities among and between personality states and situation characteristics (Rauthmann et al., in revision), (c) predicting contact and construal of situations by personality traits (Rauthmann et al., 2015c), and (d) tracking people's situations on Twitter (Serfass and Sherman, 2015). Further, Rauthmann (in press) has demonstrated how the DIAMONDS dimensions essentially capture evolutionarily important motivational processes and content. Taken together, the DIAMONDS dimensions offer a broad and useful taxonomy of the psychological characteristics of situations. As such, we will make use of this taxonomy in our empirical part later.

*Classes* (e.g., work situations, interpersonal situations, etc.) denote types or groups of entire situations with similar cue constellations (e.g., all situations with people in them may be “interpersonal situations”) or similar levels or profiles of situation characteristics (e.g., all situations which score highly on pOsitivity and Sociality may denote “pleasant social interaction situations”). The most prominent and inclusive taxonomy comes from van Heck (1984, 1989) who identified 10 situation classes: interpersonal conflict, joint working and information exchange, intimacy and interpersonal relations, recreation, traveling, rituals, sport, excesses, serving, and trading.

**Figure 2:**
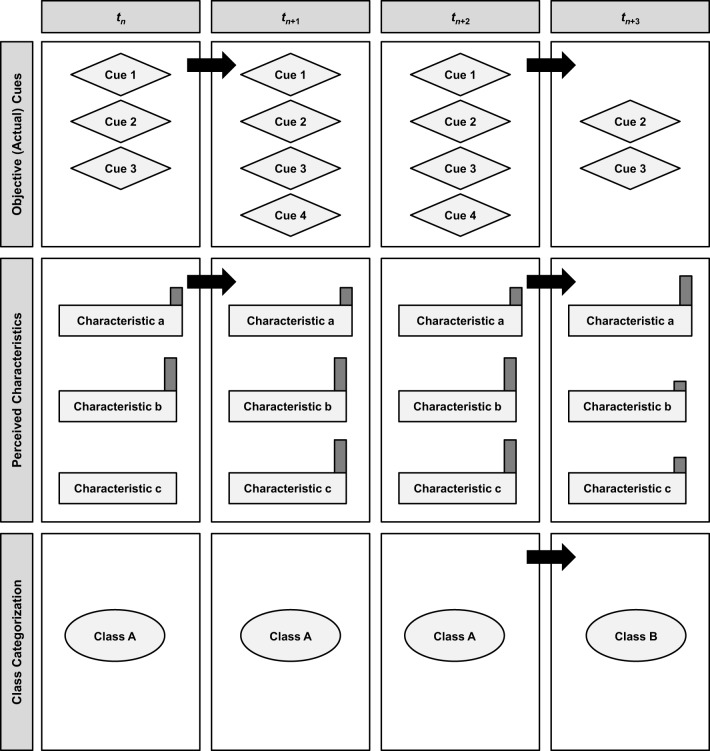
**Change of different situation variables**. Thick black arrows denote a change.

As Figure 2 summarizes, situation change may be studied according to whether (or to what extent), when, how, and why cues, characteristics, and/or classes change. Ideally, situation change would be tackled for cues, characteristics, and classes simultaneously in one design, but theory, preferences of researchers, and/or design restrictions (e.g., participant burden, financial costs, etc.) may limit the ways in which situation change is studied. Thus, we present here briefly different ways of examining situation change.

#### Change of cues

The first row of Figure 2 concerns the change of situation cues. One could think of this in a nomothetic sense (i.e., the data are averaged across many participants in a study and thus capture processes at the between-persons level) or in an idiographic sense (i.e., the data come from one participant only and thus capture within-person processes). Though cues could, in principle, also be reported by participants (e.g., by asking them which cues they noticed in their situation; see Sherman et al., 2010 for this methodology), this approach hinges upon several assumptions. First, it assumes that people can actually report objective cues. In reality though, we suspect that people are more likely to report some interpretation of cues or even characteristics. Second, this approach assumes that cues are only important if they are consciously noticed and reported. However, cues may also work outside of consciousness (i.e., be implicitly processed) and generate behavioral consequences. As such, if cues should be in the focus, then we would advise measuring them as directly as possible (not via participants)^1^. One potentially fruitful avenue for this approach lies in the use of convolutional neural networks to detect and extract cues from streams of photographs. Changes in photostreams can be used as one indicator of situation change (Bolaños et al., 2015).

For objective cues, Figure 2 shows the proceedings of 4 cues (Cues 1–4) through 4 time points (*t*_*n*_ to *t*_*n*__+3_). As can be seen, there are 3 cues (Cues 1–3) available at *t*_*n*_, 4 cues (Cues 1–4) at *t*_*n*__+1_ and *t*_*n*__+2_, and 2 cues (Cues 2 and 3) at *t*_*n*__+3_. If objective cues of situations are the benchmark criterion, then any change in objective cues denotes situation change. In Row 1, situation change then occurs from *t*_*n*_ to *t*_*n*__+1_ and from *t*_*n*__+2_ to *t*_*n*__+3_, while the situation remains stable from *t*_*n*__+1_ to *t*_*n*__+2_. Attending to the objective cues of situations allows a micro-perspective on the situation(s) studied (depending on how many cues are sampled, of course) because researchers can distinguish cues that are constantly present (Cues 2 and 3), are available only for a limited time (Cue 1), or briefly appear and disappear (Cue 4). For simplicity, we assumed in Figure 2 that cues are either present or not, thus limiting situation change to the quantity and types of cues available. However, it is also possible that (a) two or more cues “interact” with each other or form a cue conglomerate (many cues are grouped together) and/or (b) a cue changes into a different cue (i.e., change in nature). Thus, in addition to quantity, also the quality of cue changes should be examined. Studying situation change in terms of the change of objective cues in quantity and quality represents an *environment-driven approach*, and researchers must effectively strive to “catalog” the (natural or standardized laboratory) environments their participants are in. The catalog should be either exhaustive (i.e., striving to measure all quantifiable environmental information) or theory-driven (i.e., only specific cues are assessed, tailored to a specific theory or model), but not be purely *ad-hoc* (except for exploratory purposes).

#### Change of characteristics

If researchers want to emphasize more phenomenological aspects of situations, then they can focus on whether and to what extent the *psychologically important* characteristics of situations change. Row 2 of Figure 2 concerns the change of situation characteristics (Characteristics a-c). Again, such change may be examined at the within-person or the between-person level, though Figure 2 illustrates characteristics change within one individual only. As can be seen, all three situation characteristics (a, b, and c) exist in some quantity in each situation; however, the salience, importance, or relevance of each characteristic can vary at each time point. This is illustrated in Figure 2 with gray bars on top of each characteristic: the higher the bar, the more defining the characteristic is of the situation at a given time point. Thus, if psychological characteristics of situations are the benchmark criterion, then any change in the salience or the importance of situation characteristics (which are used to describe a situation) denotes situation change. As can be seen in Figure 2, the gray bars of Characteristics a, b, and c are identical at *t*_*n*__+1_ and *t*_*n*__+2_, indicating that the individual perceived those situations as psychologically identical. At *t*_*n*__+3_, however, the importance of the three characteristics shifts, such that Characteristic a now gains relatively more weight than Characteristics b and c. Thus, situation change in terms of the change of psychological characteristics would have occurred from *t*_*n*_ to *t*_*n*__+1_ and from *t*_*n*__+2_ to *t*_*n*__+3_.

The segmentation of situations according to their psychological characteristics presents a more molar approach as opposed to the molecular approach taken when examining cue changes. As such, this approach will not be as measurement-heavy as with cues, but it does require input from at least the participant(s) in the situation. This may make some researchers uncomfortable because situation characteristics now are essentially people's perceptions only (which is no problem with perceived cues because they have a real-life counterpart cue that can be physically measured). As such, a situation variable is essentially a person variable (a perception). However, Rauthmann et al. (2015a) showed that this problem can be tackled by employing multiple perceivers (or sources of ratings) when characteristics are to be rated (for empirical applications, see Sherman et al., 2010, 2012, 2013; Rauthmann et al., 2015c). In their terminology, participants physically in the situation and affected by it are termed “raters *in situ*;” bystanders or confederates in the situation but not acting or personally detached from it “raters *juxta situm*;” and laboratory assistants not in the situation and detached from it “raters *ex situ*.” Obtaining ratings from other sources than raters *in situ* grants the opportunity to derive scores shared between raters *in situ* and raters *juxta situm* and/or *ex situ* (= consensual aspects of the situation) and not shared between different raters (= idiosyncratic aspects of the situation) (see Rauthmann et al., 2015c).

#### Change of classes

At a considerably high level of abstraction, researchers may be interested whether or to what extent the class of a situation changes (not just its single cues or some set of characteristics). Situation classes can be derived in two ways. First, situation class membership can be assessed directly by asking raters *in situ, juxta situm*, and/or *ex situ* (including the researchers) to categorize the situation into a certain group or type of situations. Second, situation class membership can be assessed indirectly by grouping situations with similar (a) cues (or cue constellations) or (b) levels or profiles of situation characteristics (measured by ratings *in situ, juxta situm*, and/or *ex situ*) together. Regardless of which of these methods is used, the result is an abstract, nominal categorization of a situation to a certain class (e.g., a threat situation, a work situation, etc.). If class memberships of situations are the benchmark criterion, then any change in class membership denotes situation change. As with cues and characteristics, such class membership change may be studied between and within persons.

Row 3 of Figure 2 depicts changes in class membership. As can be seen, the situations at *t*_*n*_ to *t*_*n*__+2_ belong to Class A, while the situation at *t*_*n*__+3_ belongs to Class B^2^. Thus, situation change occurs from *t*_*n*__+2_ to *t*_*n*__+3_. (Note that this also corresponds to how cues and characteristics change as, on average, the cues and characteristic levels are at *t*_*n*_ to *t*_*n*__+2_ more similar to each other than to those at t_*n*+3_ where the situation seems to have changed markedly).

### Analytical level

As alluded to in the previous explanations of Figure 2, situation change may be examined nomothetically or idiographically. *Nomothetic analyses* concern estimates of situation change across individuals (usually for situations that are similar for the population of participants studied), allowing to examine inter-individual differences in between-person analyses. For example, interesting between-person questions are: Do some people experience more situation change than others? Are inter-individual differences in the level of neuroticism related to perceiving more frequent situation changes?

*Idiographic analyses*, on the other hand, concern the stability or variability of situations (cues, characteristics, classes) within individuals, allowing to examine intra-individual differences in within-person analyses. For example, interesting within-person questions are: How often does Alex experience adverse situations? Do such adverse situations lead to more Adversity down the road or are those situations only single (but intense) instances? Do they occur with certain regularity? Do they change into other situations (e.g., they start as adverse, but usually end pleasant)?

Ideally, situation change studies would cater to both between- and within-person questions as nomothetic and idiographic perspectives and analyses, respectively, are not irreconcilable opposites, but can be combined. For example, experience sampling or ambulatory assessment methodology (Shiffman et al., 2008), where participants report upon their current situation and mental states several times a day for several days (prompted by their smartphones or PDAs), grants the opportunity to examine real-time person-situation transactions at both a between- and within-person level (see Fleeson, 2007; Sherman et al., 2015). We believe that particularly this methodology will be quite useful in exploring and understanding situation change at different data-analytical levels.

### Analysis of change data

Methodological and data-analytic advances in analyzing Intensive Repeated Measurements in Naturalistic Settings data (Moskowitz et al., 2009) will likely be the most fruitful way of studying situation change *in vivo*. Consensus about when change occurs can be examined qualitatively via subjective impressions of change points (e.g., by asking raters *in situ* and/or *ex situ* when a situation has changed). However, a more convincing case for consensus on situation change can be made by not only examining consensus on whether a situation has changed, but also by assessing how and to what degree the situation has changed. This will be best achieved by approaches that quantify characteristics of situations. As such, situation change can also be quantitatively assessed by determining to what extent (*in situ, juxta situm*, or *ex situ*) ratings of the psychological characteristics of the same situation correlate higher with each other than ratings of the psychological characteristics of different, but temporally adjacent, situations. Quantity of situation change, for each individual, can be measured at the level of a single situation characteristic or at the level of profiles. To measure the former, one could compute the within-person standard deviation (*SD*) of each DIAMONDS situation characteristic (rated either *in situ, juxta situm*, or *ex situ*) across time (see Fleeson, 2001, 2007 who quantified variability in personality expressions and situation characteristics across time like this). To measure the latter, one could correlate the DIAMONDS profile scores for one situation with the DIAMONDS profile scores for another (or all other) situation(s). Such profile correlations reflect “situational similarity” (Sherman et al., 2010); low(er) profile correlations would reflect strong(er) differences in situations across time. We can then attempt to explain both of these measures of average situation change via correlation/regression with personality or changes in momentary states (Question 2). Lastly, average situation change can be used as a predictor of outcomes such as momentary personality, affect, or self-esteem (Question 3). Beyond these rather simple analyses, situation change can also be modeled using more advanced techniques. For example, differential equation modeling (Deboeck, 2011) can be used to identify within-person, non-linear patterns of situation change (e.g., oscillation) over time, and the nested nature of the data (situations within participants) will require, for some questions, multilevel models (Nezlek, 2012) or autoregressive models (Eid et al., 2012).

### Empirical examples

Below, we present some findings from preliminary data where we demonstrate different data-analytical procedures of studying situation change. First, we quantify situation change as the consensus between different raters on when a situation has changed. Second, we zoom in on situation change by examining how much characteristics change. We perform these two analyses for one individual only to demonstrate an idiographic approach. After that, we perform different analyses on a data set with *N* = 60 participants to demonstrate a nomothetic approach. First, we examine mean-level change of situation characteristics for two persons only (to demonstrate individual differences). Second, we quantify change at the level of single situation characteristics (within-person *SD*s) and characteristic profiles. Lastly, we illustrate how the relationships among situation characteristics may change across situations in dynamic network analyses. All data were gathered in accordance with the United States Department of Health and Human Services code of federal regulations title 45, part 46 (45 CFR 46) and approved by the Florida Atlantic University Institutional Review Board. All subjects gave written informed consent in accordance with the Belmont Report.

#### Data set #1: agreement on situation change

Determining the existence of psychological phenomena (e.g., personality) is a much more difficult task than determining the existence of physical objects (e.g., other people, a book, a cake) because psychological constructs lack concrete physical existence. However, using the time-honored practice of *consensus*, psychology has had no trouble demonstrating the real and meaningful existence of a large number of psychological constructs. We propose that the same practice can be used to determine the existence of situation change: if people reasonably agree that a situation has changed, then we can say with probabilistic certainty that it has indeed consensually changed.

**Figure 3:**
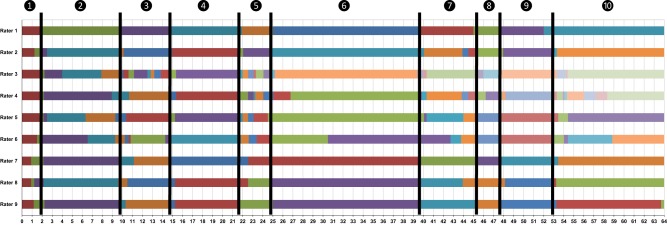
**Situation changes according to 9 independent judges (Data set #1)**. Change in colors from left to right represent each rater's individual change point. Black bars represent consensual change points. Time (in minutes) is depicted along the *x*-axis. Situation descriptions: (1) Taking dogs outside; (2) Watching dogs come back in; (3) Feeding dogs; (4) Driving to work; (5) Stop at coffee shop for coffee; (6) Continue driving; (7) Walk into office; (8) Sitting in office; (9) Walking to class; (10) In class.

In a pilot project aimed at examining this hypothesis, we had one participant wear a mini-video camera (about the size of a thumb) on his shirt from the moment he woke up for a little over an hour. We then enlisted nine research assistants to independently view the recorded video. They indicated, based on their own subjective interpretations, each time the situation the participant was in changed. The results from this task are displayed visually in Figure 3. As can be seen, the raters differed in their perceptions of whether or not a situation changed (e.g., Rater 3 indicated more frequent changes than Rater 9), as indicated by a change in color in Figure 3. However, raters also demonstrated approximate consensus about when situation change occurred, as indicated by the vertical black bars. From these ratings, it appears that the individual wearing the mini-video was in approx. (at least) 10 different situations (or situational episodes). Thus, we suspect that situation change is indeed a real phenomenon that can be detected by others with reasonable amounts of consensus. However, this exercise only treats situation change as a binary phenomenon (a situation has either changed or not) and does not allow us to delve into the more substantive questions of why a situations has changed or which aspects have changed.

#### Data set #1: change of characteristics

**Figure 4:**
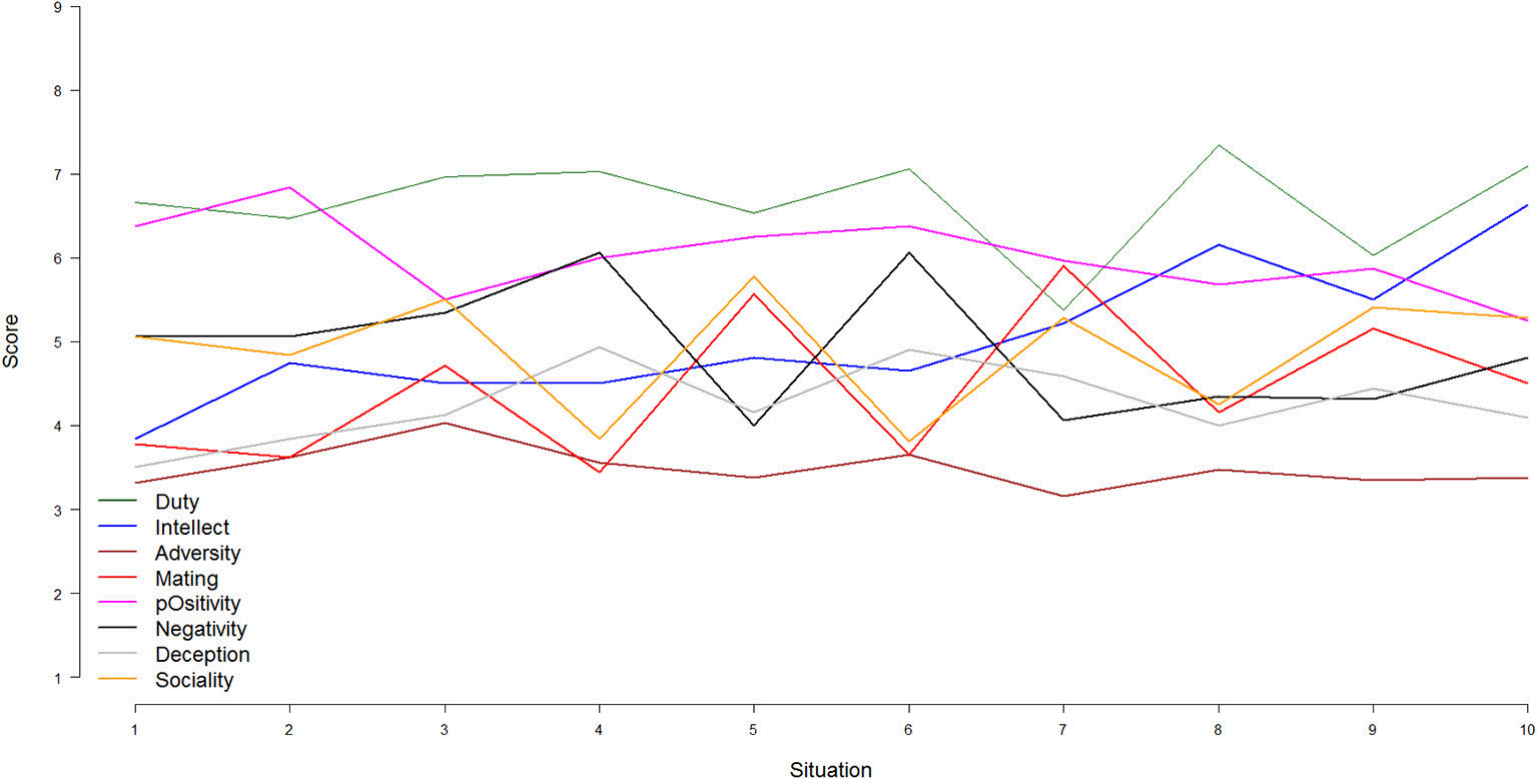
**Mean-level changes of the Situational Eight DIAMONDS over a 1 h period (10 situations) of one participant (Data set #1)**. Duty: green; Intellect: blue; Adversity: brown; Mating: red; pOsitivity: magenta; Negativity: black; Deception: gray; Sociality: orange. Situation descriptions: (1) Taking dogs outside; (2) Watching dogs come back in; (3) Feeding dogs; (4) Driving to work; (5) Stop at coffee shop for coffee; (6) Continue driving; (7) Walk into office; (8) Sitting in office; (9) Walking to class; (10) In class.

To quantify situation change with respect to a more fine-grained analysis of situation characteristics in our pilot video, we sampled two separate 30 s clips from each of the 10 different situations indicated between the black bars in Figure 3 (i.e., 20 total clips). Two groups of research assistants (*n* = 4 in each group) then watched one of the 30 s clips from each of the 10 situations (clip order was counter-balanced) and rated the psychological characteristics of the situation shown in those clips using the RSQ-8 (32 items; Rauthmann et al., 2014). The participant's (rated) DIAMONDS characteristics are plotted in Figure 4. As can be seen, the participant's situations were relatively high on Duty (while Adversity and Deception were low), and Intellect gradually increased across situations (as the person approached school and eventually arrived in class).

If the black bars noted in Figure 3 represent actual situation change, then we would expect to find that the 30 s clips from the same situation are rated as more psychologically similar than 30 s clips from different situations. This is indeed what we found. Specifically, ratings of the same situation were more similar to each other (average *r* = 0.35) than ratings of different situations (average *r* = 0.22). Such results suggest that people are sensitive to situation change and that the RSQ-8 may be used to identify situation change concerning psychological characteristics. Of note, this result also indicates that the pilot participant's situations showed some stability across time (*r* = 0.22), which will be addressed shortly.

The ability to quantify situation change is crucial for this research because it allows us to investigate further questions such as: (1) How much situation change does a person experience across the day? (2) How consistent is change across time (e.g., hours, days)? (3) How much within- and between-person variance is there in situation change? These questions can be addressed at both the level of a single situational characteristic (e.g., How much does a person's experience of Duty change across time?) and of the situational profile (e.g., How stable/variable is profile of situation characteristics that a person experiences across time?). As noted, the answer to this last question for the pilot participant was *r* = 0.22. This finding suggests that, while there was some stability in this person's situational experience over time, there was also a great deal of variability. Such variability can be visualized, as done in Figure 4, which shows average coder ratings of the Situational Eight characteristics in each situation. As can be seen, there was a large amount of variability in the pilot participant's situation characteristics across time. Further, some situation characteristics showed more variability than others. Adversity (green line) was relatively low and stable across time for this participant. Intellect (red line) showed more variability and generally increased over time (which is nice to see because the 10th situation was in a college classroom). Lastly, in terms of their overall Situational Eight profiles, Situations 1 and 2 look more similar to each other than Situations 7 and 8.

#### Data set # 2: inter-individual differences in situation change

So far, we have demonstrated how situation change could be studied for one individual with idiographic analyses. However, many psychologists may be interested in how situations change generally or in comparing situation change between different individuals (see Dalal et al., 2015 for a review). To this end, we ran a follow-up study with *N* = 60 participants (undergraduate students) who now wore mini-video cameras for 24 h. Participants were asked to record, for approx. 30s, each new situation they encountered (this time we allowed them to use their own definition of what constituted a new situation). These videos were later rated by 4 research assistants on the Situational Eight DIAMONDS situation characteristics with two items per dimension from the RSQ-8 (Rauthmann et al., 2014) for economic reasons. We then formed aggregate scores of the DIAMONDS for each situation (across the 4 research assistants). For illustrative purposes here, we chose two individuals with more than 10 situations sampled: For showing differences in mean-level changes, we selected Subject 29 (19 situations) and Subject 30 (10 situations).

**Figure 5:**
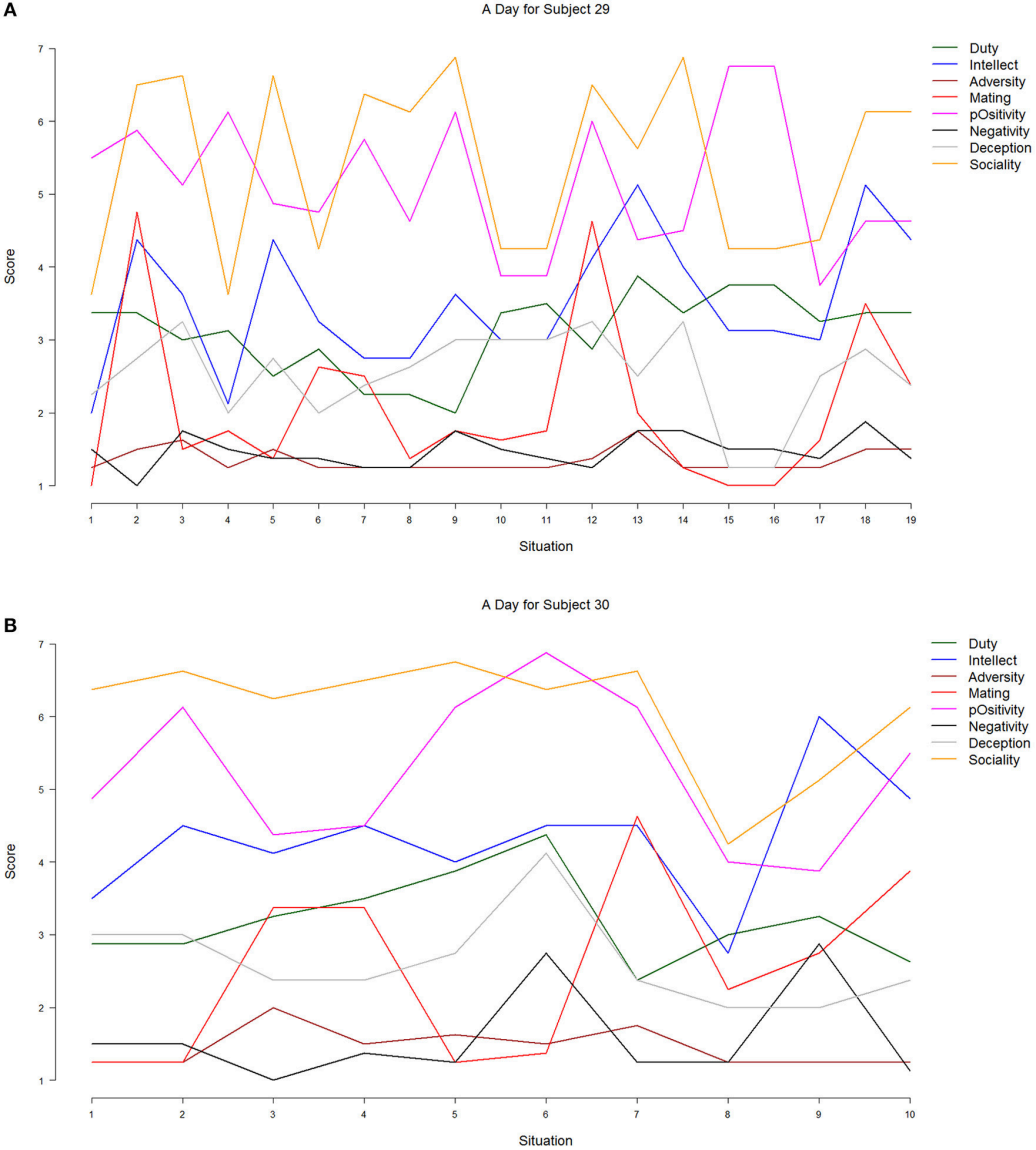
**Mean-level changes of the Situational Eight DIAMONDS for two participants (Data set #2)**. Duty: green; Intellect: blue; Adversity: brown; Mating: red; pOsitivity: magenta; Negativity: black; Deception: gray; Sociality: orange. **(A)** Subject 29, **(B)** Subject 30.

We simply plotted the DIAMONDS composite scores across the respective situations from Subjects 29 and 30 (see Figure 5) to get a picture of inter-individual differences in mean-level changes (as in Figure 4). As can be seen, there were commonalties and differences between both participants. As for the commonalities, the situations of both participants could be characterized, on average, as more social and positive than deceptive, adverse, and negative. This is consistent with other research finding that the typical situation, even across different countries, is mildly positive and social (Guillaume et al., 2015). However, there were also differences between both participants. Subject 29's situations seem to change more strongly than Subject's 30s; they showed more mean-level changes across different situation segments. This may be a first hint at inter-individual differences in the *degree* of situation change.

#### Data set # 2: single- and profile-level analyses

**Figure 6:**
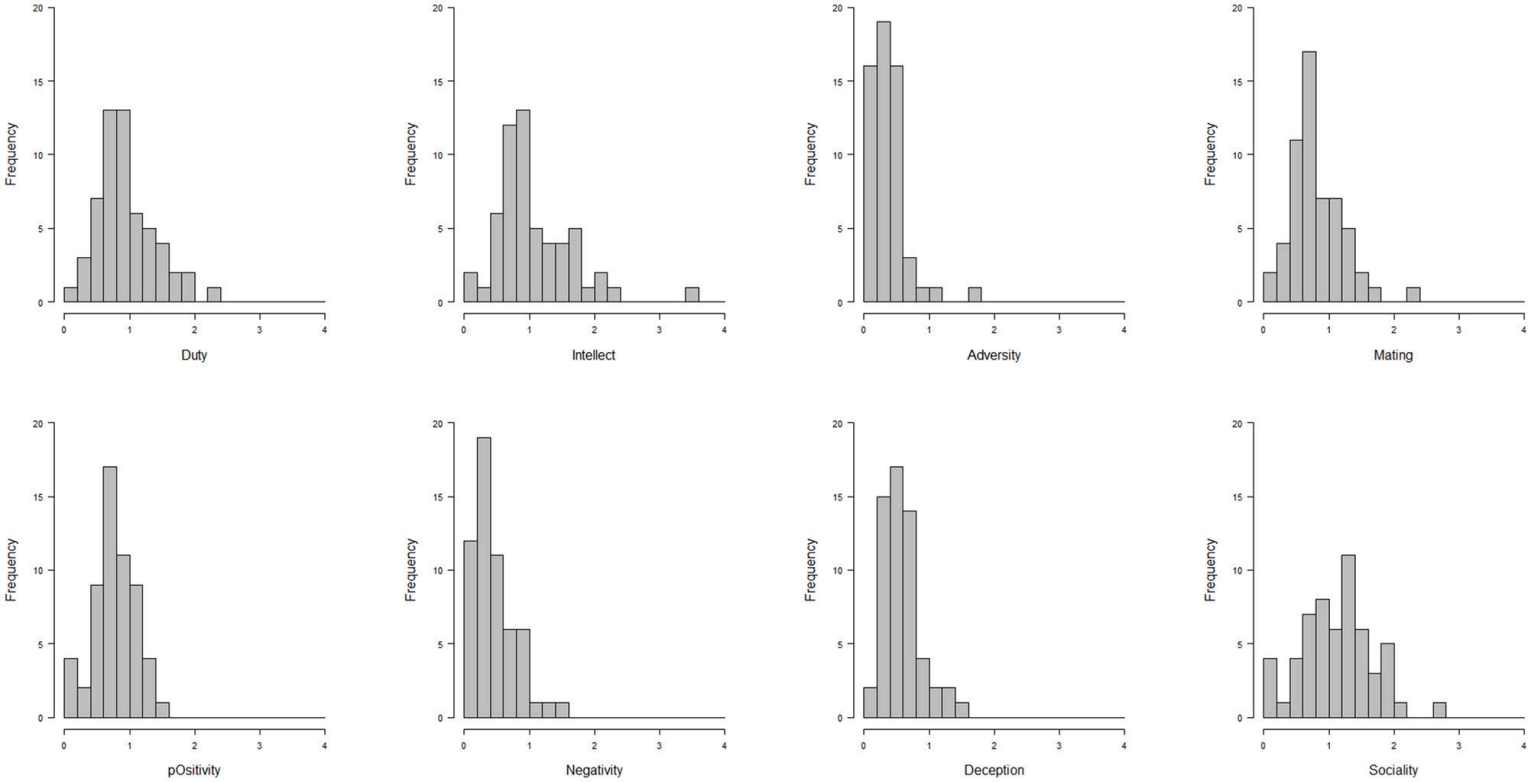
**Density distributions of within-person *SD*s in the Situational Eight DIAMONDS (Data set #2)**.

Situation change in terms of variations in situation characteristics can be analyzed for each single characteristic or for a profile of characteristics. At the single characteristics level, the within-person *SD* (across all situations) indexes the amount of change. As can be seen in Table 1, Sociality and Intellect showed, on average, the most variation, while Adversity and Negativity the least. Figure 6 additionally shows the density distributions of within-person *SD*s for all DIAMONDS. As can be seen, there are sizeable individual differences in Duty, Intellect, and Sociality, while there are less in the other characteristics dimensions. This inter-individual variation could, at some point, be explained by other individual difference variables, such as self- or peer-reported personality of participants.

**Table 1:** **Descriptive statistics of within-person SD of situation experiences**.

**Dimension**	***n***	***M***	***SD***	**Median**	**[min to max]**	**Skewness**	**Kurtosis**	***SE***
Duty	57	0.94	0.44	0.88	[0.00–2.30]	0.77	0.45	0.06
Intellect	57	1.06	0.59	0.90	[0.00–3.45]	1.28	2.90	0.08
Adversity	57	0.37	0.28	0.35	[0.00–1.65]	2.08	7.02	0.04
Mating	57	0.80	0.41	0.70	[0.09–2.30]	1.10	1.80	0.05
pOsitivity	57	0.76	0.31	0.69	[0.00–1.42]	−0.21	−0.40	0.04
Negativity	57	0.46	0.32	0.40	[0.00–1.50]	1.04	0.66	0.04
Deception	57	0.57	0.30	0.54	[0.00–1.50]	0.88	1.05	0.04
Sociality	57	1.11	0.55	1.16	[0.00–2.74]	0.14	0.20	0.07

n = 57 (from N = 60) because 3 people only recorded 1 situation. Within-Person SD = within-person standard deviation.

**Figure 7:**
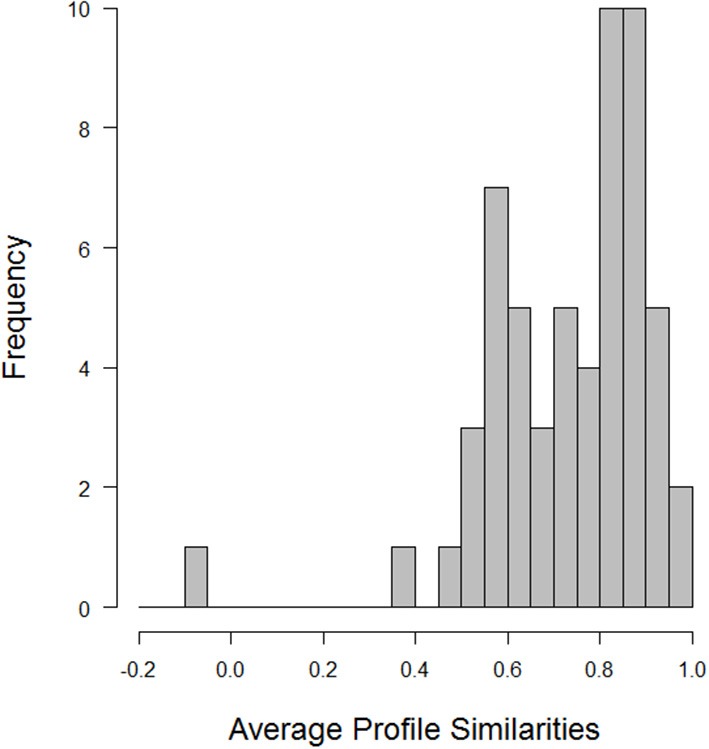
**Density distribution of average profile similarities**.

At the profile level, the correlation between a profile of characteristics in one situation and the profile in the next situation indexes situational similarity or stability (see Sherman et al., 2010). One can then compute such correlations for all pairs of situations and average the profile correlations for each person. The grand average profile similarity across all participants was 0.79 (median = 0.79, *SD* = 0.44; min = −0.06, max = 1.00), indicating that Situational Eight DIAMONDS profiles remained, on average, relatively stable within persons. However, as Figure 7 (histogram of average within-person profile similarities for all pairs of situations) shows, there were also relatively large individual differences in average situational similarities. This suggests that, for some people, there is more, and for others less profile stability (i.e., they show more severe changes in situational experiences). These individual differences could, again, be explained by other individual differences variables (e.g., personality) at some point.

#### Data set # 2: dynamic networks of situation change

The preceding analyses are fairly static and do not readily allow inferences about temporal dynamics of the interrelations between situation characteristics. Thus, we used network analyses to examine processes of situation change. This involved several steps: For Subjects 55 and 58 (those with the largest numbers of situations sampled), we (a) within-person centered the DIAMONDS composite scores for each situation, (b) stored each of those as a matrix (with 1 row and 8 columns), (c) multiplied that matrix by its transpose to create a matrix of cross-products (treating this matrix of cross-products as a similarity matrix), and (d) repeated Step c for each consecutive pair of situations (i.e., each situation transition). We then were able to model these data with the R package “qgraph” (Epskamp et al., 2012; see also Costantini et al., 2015) as a network, consisting of the Situational Eight DIAMONDS, across the situations of the participants. In these networks, the arrows represent temporal associations from *t*_*n*_ to *t*_*n*_ + 1 (i.e., how prior pOsitivity predicts later Sociality and so on). Figure 6 shows gif-animated networks of how the Situational Eight DIAMONDS characteristics change across situations for Subject 55 (27 situations) and 58 (23 situations). Changes are between adjacent situations only (e.g., a participant's Situation 1 to his/her Situation 2, Situation 2 to 3, and so on). Red arrows reflect negative associations and green arrows positive associations; thicker arrows mean stronger (positive or negative) associations. Note that we have modeled the change of *relationships* among and between the DIAMONDS in the network animations.

**Figure 8:**
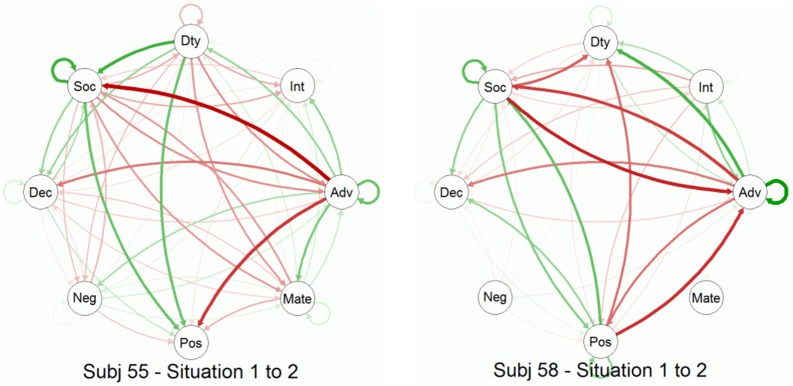
**Animated situation change networks of two participants across time**. Situation change networks are animated GIFs. Red arrows: negative prediction; green arrows: positive prediction. Dty: Duty; Int: Intellect; Adv: Adversity; Mate: Mating; Pos: pOsitivity; Neg: Negativity; Dec: Deception; Soc: Sociality. The animated gif forms can be found in the Supplemental materials.

**Figure 9:**
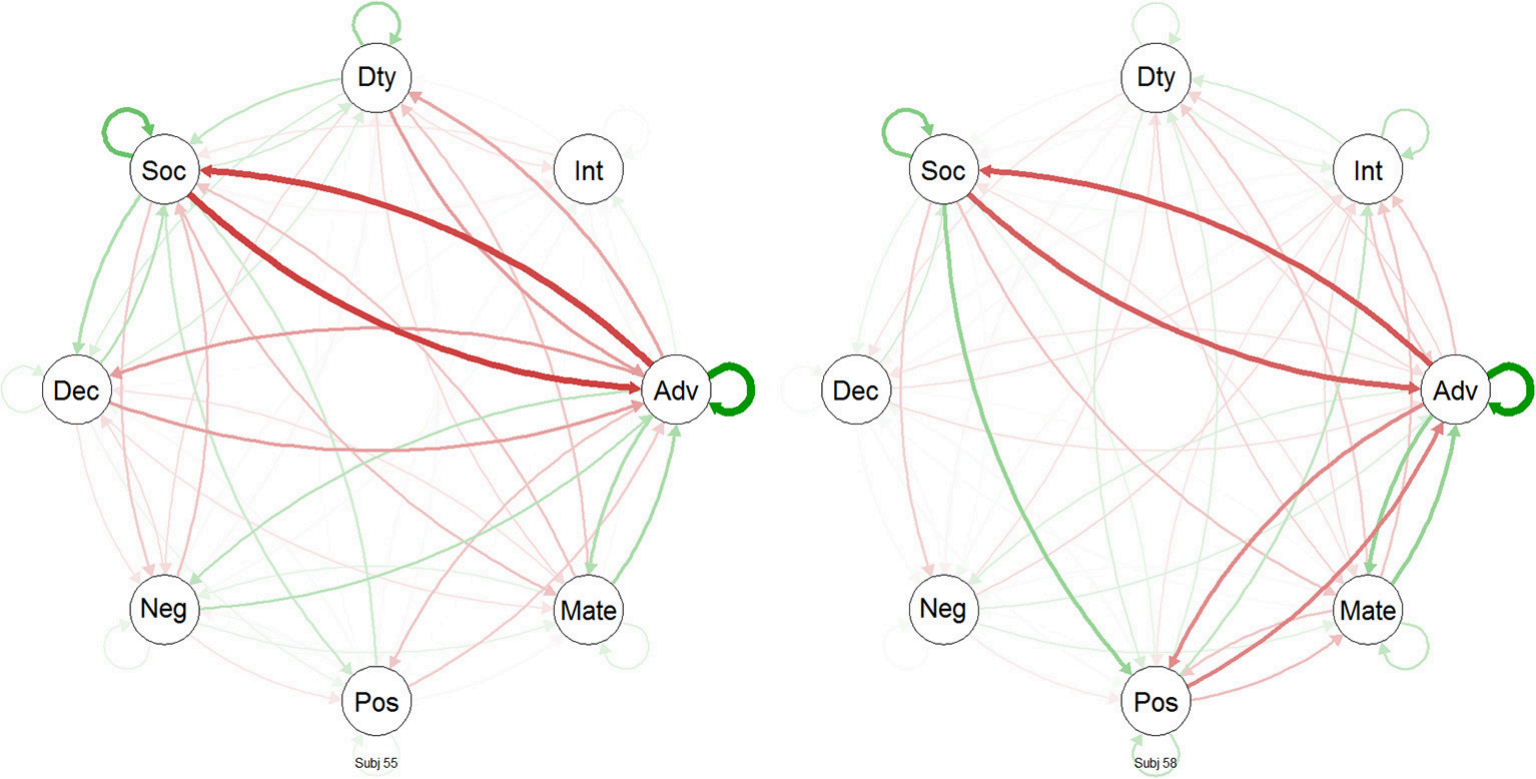
**Average situation change networks of two participants**. The networks represent the averages across all situation-to-situation changes from Figure 6. Red arrows, negative prediction; green arrows, positive prediction. The.avi video clip “AvgChangeForEachSubject” in the online Supplemental Materials shows average situation change networks for all participants. Dty: Duty; Int: Intellect; Adv: Adversity; Mate: Mating; Pos: pOsitivity; Neg: Negativity; Dec: Deception; Soc: Sociality.

As can be seen in Figure 8, when only looking at transitions from Situation 1 to Situation 2, there were again commonalities and differences between the change networks of Subjects 55 and 58. For example, both participants had in common that prior Adversity predicted less later Sociality. However, there were also differences. For example, prior pOsitivity predicted more later Duty for Subject 55, while it was less for Subject 58. Indeed, Adversity was generally more “active” in Subject 58's change network: It predicted more later Duty and less later Sociality and it was predicted by less prior Sociality and pOsitivity. Because both participants were not in the same situation but in different ones, the apparent differences found here may be spurious: Both participants could actually be fairly similar, but their situations are just actually different. To account for this explanation, we also computed the average situation change networks of Subjects 55 and 58 (see Figure 9). As can be seen, the inter-individual differences were not as pronounced once we examined *average* change, though they did not disappear. For example, Adversity still had a more prominent role in Subject 30's network.

To get a glimpse of how strong inter-individual differences of intra-individual situation change networks were, the online Supplemental Materials contain an .avi video clip “AvgChangeForEachSubject” that depicts the average situation change networks for all participants. Note, however, that the number of situations differed substantially between participants (*M* = 9.62 different situations, *SD* = 6.19, min = 1, max = 27).

## Explaining situation change

If situation change exists and if it can be quantified, the next important question is determining the factors that might explain, or at least be associated with, situation change: *Why* do situations change? Our pilot findings described above indicated that there was both consistency and variability of situation experiences and change across time. Though empirical literature has almost nothing to say about situation change, there is good reason to expect that stable personality dimensions will be associated with situation change. For example, experience sampling research assessing momentary affective states has shown that individuals high in Neuroticism are more likely to experience dramatic shifts in affect (Eaton and Funder, 2002). Thus, we would anticipate that individuals high in Neuroticism also experience more variability in their situations over time. Not only focusing on stable personality dimensions, we might also anticipate that changes in momentary personality expressions (Fleeson, 2001, 2007) or transient goals (e.g., what a person needs, wants, desires, or intends in a given situation) correspond with situation change. Additionally, person-situation transactions may help explain situation change.

### Person-situation transactions: situation management strategies

Many situations seem to “simply change on their own,” but generally people can also influence their situations in different ways; they are not merely passively or randomly “exposed” to situations, but also shape and define them (Plomin et al., 1977; Buss, 1981, 1987; Scarr and McCartney, 1983; Snyder and Ickes, 1985; Ickes et al., 1997; Caspi and Roberts, 2001). Table 1 gives an overview of six possible person-situation transactions, which we refer to as *situation management strategies*, that allow people to “manage” situations by experiencing or shaping situations (differently than before). Situation management strategies refer to how people deal with, navigate in, and govern their daily situations and can thus explain situation change. Broadly, such management can be voluntary (≈ explicit, conscious, intentional, deliberate, effortful, systematic) or involuntary (≈ implicit, unconscious, unintentional, indeliberate, effortless, capricious). To better contrast the different strategies in Table 2, they are evaluated in terms of (a) intentionality of utilizing the strategy, (b) effort for the strategist, (c) control granted to the strategist, and (d) (physical) activity of the strategist while pursuing the strategy (Table 3).

**Table 2:** **Situation management strategies in extant literature**.

**Person-situation transactions: situation management strategies**	**Scarr and McCartney (1983): genotype → environment effects**	**Buss (1987): person-environment correspondence processes**	**Caspi and Roberts (2001): person-environment transactions**
Construal	?	?	Reactive
Maintenance	(Passive)	?	?
Evocation	Evocative	Evocation	Evocative
Selection	Active	Selection	Pro-active
Modification	?	Manipulation	?
Generation	Active	Manipulation	?

Terms from the authors (in columns) were matched with the six situation management strategies. Parentheses () mean that the term may probably describe the respective strategy. Question marks (?) mean that there is probably no direct analog.

**Table 3:** **Overview of major types of important situation management strategies**.

**Strategy**	**Individual difference variables**		**Properties**			
	**Situation …**	**Individual differences in the tendency to …**	**Intentionality**	**Effort**	**Control**	**Activity**
Construal	Constructor	Uniquely construe situations differently from the consensus	/	/	++	−−−
Maintenance	Sustainer	(Passively) remain in a situation without changing it, thereby possibly maintaining it	/	/	−−	−−
Evocation	Conjurer	(Unwillingly) elicit certain situations	−−−	−−−	−−	/
Selection	Picker	(Willingly) select certain situations (without creating them)	+	/	++	+
Modification	Engineer	(Actively) modulate situations in a certain goal-serving way	+++	++	++	++
Generation	Creator	(Pro-actively) create situations in a certain goal-serving way	+++	+++	+++	+++

+++: extremely strong; ++: very strong; +: strong; /: strong to weak; −: weak; −−: very weak; −−−: extremely weak. These distinctions are only approximations (by the two authors of this work) and will need to be empirically challenged with real data.

#### Construal

People may distinctly perceive situations differently from how other people see them. We refer to this strategy as *situation construal*, and there may be individual differences in the extent to which people are situation constructors. Construal can be intentional and effortful (e.g., during cognitive restructuring mechanisms) or unintentional and automatic (e.g., because of motives and values, but also psychopathology). Construing situations in a certain manner (e.g., trying to find the silver lining in an otherwise dire situation) may grant the situation constructor at least cognitive control over the situation by changing it in his/her unique perceptions. Because construal resides only at the mental level, no physical activity is involved.

#### Maintenance

People may remain in and maintain a situation, thus fostering the stability of a situation and consequently inhibiting change. We refer to this strategy as *situation maintenance*, and there may be individual differences in the extent to which people are situation sustainers. Maintenance can be intentional (especially while bearing or sitting out a situation) or unintentional. Depending on the characteristics of the situation, it may require effort to remain in the situation or not. Maintaining a situation should usually not result in much active control, except if the *status quo* needs to be upheld against change (e.g., if one wants a situation to stay as it is, but other parties want change). The strategy is marked by passivity although active resistance may be used to achieve maintenance of an already existing situation (to preserve it as it is). To our knowledge, maintenance has so far not been sufficiently conceptually addressed in traditional transaction models (e.g., Buss, 1987).

#### Evocation

People may engender certain situations without specific intentions of doing so^3^. We refer to this strategy as *situation evocation*, and there may be individual differences in the extent to which people are situation conjurers. Situation evocation captures genuinely unintentional elicitations of situations (e.g., when one's behavior triggers reactions of others, thus changing the situation). Accordingly, usually no effort has been invested in bringing about the elicited situation because the situation was neither planned nor intended. As a result, the situation conjurer only has limited options to control the inadvertent situation, and he/she may be active or not during the evocation process^4^.

#### Selection

People may choose (i.e., approach or avoid) certain situations. We refer to this strategy as *situation selection*, and there may be individual differences in the extent to which people are situation pickers. Selection is usually an intentional process (e.g., thinking about where to go), but situations may also not be explicitly sought because (a) situations can traverse “naturally” into different situations (e.g., another person joins and the situation changes), (b) people more or less “just go with the flow” instead of deliberately selecting every new situation to engage in, and (c) people can only select situations within the limits of a given pool of possible situations to choose from. As such, the effort in choosing situations may be more or less, depending on whether a situation is intentionally sought after (e.g., a romantic date) or unintentionally just happens (e.g., a stimulating conversation). However, since intentional selection includes not only the promotion but also the avoidance of certain situations, this strategy allows the utilizer a certain amount of control and requires some level of activity.

#### Modification

People may actively change an existing situation into something different (e.g., in a goal-serving way). We refer to this strategy as *situation modification*, and there may be individual differences in the extent to which people are situation engineers. Modification differs from selection in that not a new or qualitatively different situation is sought, but an already existing one actively “worked on” and transformed. As such, it harbors a high degree of control and activity, relatively to the other strategies. Modification also differs from evocation in that modulations are conducted intentionally and with some amount of effort (time, energy, etc.).

#### Creation

People may pro-actively and purposefully create new situations in the service of their goals. We refer to this process as *situation creation*, and there may be individual differences in the extent to which people are situation creators. Creation differs from modification in that not a pre-existing situation is transformed, but an entirely new one willingly created. As such, the creation strategy harbors, relative to all other strategies, the highest levels of intentionality (creation is purposeful and goal-oriented), effort (creation requires resources), control (creation implies control over the creative process), and activity (creation requires work).

### Different types of situation change through different situation management strategies

Taking the previous explications into account, we can now ask, for each person, to what extent the situation (i.e., cues, characteristics, classes) changed because (a) he or she perceived it differently (*construal change*), (b) it was changed by something outside of his or her control (*evocative change*), (c) he or she left for another situation (*selective change*), (d) he or she actively changed it (*manipulative change*), or (e) he or she created an entirely new situation (*generative change*)? By categorizing situation changes in this manner we can more specifically assess the associations between types of change (construal, evocative, selective, manipulative, generative) and personality and momentary states. For example, questions to be asked then include: (1) Are certain personality dimensions associated with a tendency toward particular kinds of situation change? (2) Does the presence of particular goals or affective states predict different kinds of situation change?

It can be a difficult task disentangling which strategy (Table 2) has been used by a person and, by extension, which type of situation change has occurred, but we believe that each strategy leaves characteristic “traces” in how strongly and fast situations are changed within a person. We refer to these traces as *flux functions* which describe the continuous change of situations within an individual over a certain time span. Thus, situation change can be examined in response to different situation management strategies.

**Figure 10:**
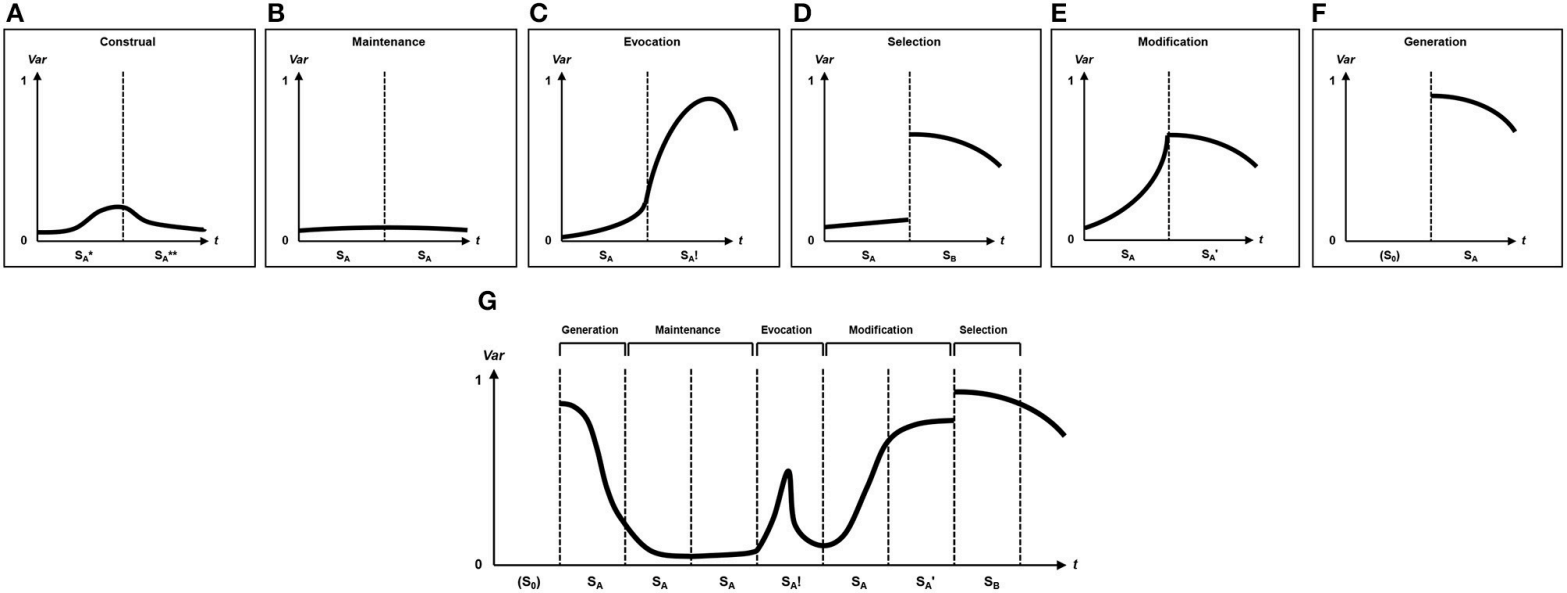
**Illustration of six situation management strategies and their possible trajectories (“flux-functions”)**. *x*-axis: Different situations along time (*t*); *y*-axis: Variability (*Var*) of the environment (0 = *total stability*, 1 = *no stability at all* = *maximal variability*). Bold lines illustratively denote the extent to which the environment is malleable, plastic, or variable. No actual data were used. Construal **(A)**: The experience of a psychological situation S_A_^*^ transitions into the construed psychological situation S_A_^**^. Maintenance **(B)**: The situation S_A_ is maintained as situation S_A_ through time. Evocation **(C)**: The situation S_A_ changes into the situation S_A_! via involuntary/inadvertent evocation. Selection **(D)**: The situation S_A_ is de-selected (avoidance), and the situation S_B_ selected instead (approach). Modification **(E)**: The situation S_A_ is voluntarily/intentionally modified into the situation S_A_′. Generation **(F)**: The situation S_A_ has been created. Complex concatenation of strategies **(G)**: The situation S_A_ is created which is, for some time, maintained as situation S_A_ until the situation S_A_! has been evoked which is then modified into the situation S_A_′. The modified situation S_A_′ is then deselected, and situation S_B_ selected.

Hypothesized (but fictitious) flux functions are presented in Figure 10 for each strategy. The *x*-axis represents time and shows for Figures 10A–F two different situations. The *y*-axis represents variability of the actual or perceived environment (with 0 denoting total stability and 1 denoting maximal change). Figure 10A illustrates construal (construal situation change) where the experience of a psychological situation S_A_^*^ transitions into the construed psychological situation S_A_^**^. Figure 10B illustrates maintenance (which does not yield situation change, but stability) where the situation S_A_ is maintained as situation S_A_ through time. Figure 10C illustrates evocation (evocative situation change) where the situation S_A_ changes into the situation S_A_! via involuntary/inadvertent elicitations. Figure 10D illustrates selection (selective situation change) where the situation S_A_ is de-selected (avoidance), and the situation S_B_ selected instead (approach). Figure 10E illustrates modification (manipulative situation change) where the situation S_A_ is voluntarily/intentionally modified into the situation S_A_′. Figure 10F illustrates Generation (creative situation change) where the new situation S_A_ has been created. Lastly, Figure 10G illustrates a complex concatenation of strategies (showing all types of situation change) where the situation S_A_ is created which is, for some time, maintained as situation S_A_ until the situation S_A_! has been evoked which is then modified into the situation S_A_′. The modified situation S_A_′ is then deselected, and situation S_B_ selected. To our knowledge, no empirical study has so far examined any form of flux function so that this approach represents a novel avenue for future research.

### Zooming in on the processes of change

We should try to dig further and inquire about the underlying processes of situation change: *Why* do people change situations? It is likely that motivational processes (such as goals, needs, and motives) play a key role here (see Yang et al., 2009). People's goals may not only shape the way they perceive situations (Rauthmann, in press), but also how they respond to them. According to Yang et al. (2009), situations may be understood in terms of their goal content and their goal processes. Regarding content, evolutionarily important goals may be particularly important (see Brown et al., 2015) as recurring ancestral presses have likely attuned our perceptual systems to motive categories that historically fostered survival and reproductive fitness in the environment of evolutionary adaptedness. Regarding processes, what is happening or could happen to people's goals is important: Can they be achieved or are they blocked? Empirical studies (e.g., Edwards and Templeton, 2005; Yang et al., 2006) lend support to the idea that people broadly perceive situations in terms of whether they foster or hinder goal pursuit and attainment. Situations may change, in part, because people change their momentary goals, intentions, and strategies. This is also in line with recent theory and research that emphasizes the role of social-cognitive mechanisms behind the manifestation of personality traits into personality expressions (Fleeson, 2012; Fleeson and Jayawickreme, 2015). Because personality expressions and concurrent situation characteristics are intertwined (Sherman et al., 2015; Rauthmann et al., in revision), it is plausible that situation change can be similarly predicted by goal processes as can be personality expressions (e.g., McCabe and Fleeson, 2012). Taken together, attending to people's enduring and momentary goals (that are activated and salient in a given situation) should be fruitful because they may be able to illuminate why (i.e., for what reasons and for what anticipations of outcomes) people attempt to maintain or change a situation in the first place.

## Trajectories and outcomes of situation change

What are the outcomes and consequences of situation change? If situation change can be quantified (Question 1) and categorized and explained (Question 2), it becomes reasonable to ask about the consequences of situation change. Generally, effects of situation change may manifest at short-, middle-, and longer terms (see Figure 1).

For short-term consequences, we can ask: What kinds of behaviors are enacted as a result of (different kinds and magnitudes of) situation change? For example, we would expect that transitioning from a situation characterized by low Duty (e.g., there is no work to be done) to one that is high in Duty (e.g., work needs to be done) would result in a person expressing more conscientious behavior (e.g., organizing, working hard). To the extent that this person can be characterized and also describes him- or herself as a generally conscientious person, this person may experience authenticity because of increased personality-behavior fit (cf. Jones et al., under review). Additionally, the person may experience mild positive affect, self-esteem, and self-efficacy in dealing with the conscientiousness-affording situation because there is personality-situation fit (Rauthmann, 2013). Lastly, a person with appropriate responses to a situation, or behavior-situation fit, may be said to be well-adjusted to his or her surroundings and thus also garner positive social consequences (e.g., respect, reputation, more pay, etc.). Thus, situation change may stand in the service of short-term personality-behavior, personality-situation, and behavior-situation fit, and all three types of fit may entail middle- to long-term intrapersonal (e.g., affect, self-esteem) and interpersonal (e.g., status, popularity) adjustment. For example, via habitual (= typical and repeated) situation changes people may be able to cumulatively “optimize” their surroundings according to their needs and personalities. Thus, in the long haul, short-term situation changes may stand in the service of long-term developmental regulation (Haase et al., 2013) where people actively manage their surroundings and development (Baltes, 1997; Roberts and Caspi, 2003). For example, the corresponsive principle (Roberts, 2005, 2006; Roberts and Wood, 2006) specifies that (a) people modulate their situations and environments according to their traits (see Gosling et al., 2002, 2008 for personality-manifestation in personal environments) and that (b), in turn, these traits are consolidated by the selected situations and environments (e.g., via socialization processes). Thus, particularly developmental psychologists and researchers interested in personality development may attend to understanding situation change processes better.

Nonetheless, there are also several other interesting questions, such as: (1) To what extent does overall situational variability (a lot vs. little change) impact how individuals are feeling, thinking, and behaving? (2) How do individuals adjust their goal strivings as a result of situation change? (3) Does personality moderate the associations between situation change and these outcomes? Answers to these questions will provide a greater understanding to two of psychology's most important outcomes: Why do people behave the way they do, and what makes a person feel good or bad?

## Summary and conclusion

There are many ways in which situation change can be studied, depending on (a) the resolution of interest (situation—episode—environment—context), (b) the situation variables used as benchmark criteria for change (cues—characteristics—classes), (c) the measurement of situation variables (e.g., actual—perceived; *in situ*—*juxta situm*/*ex situ* rated; -oriented), (d) the level of analysis (between-person—within-person, variable-oriented—profile), and (e) the type of situation change studied (construal—evocative—selective—manipulative—generative). We hope that this article could make researches aware of this diversity and alert to important questions as well as intriguing ways of answering them. Situation change remains as of yet an overlooked concept that can enrich personality, social, and developmental psychology.

### Conflict of interest statement

The authors declare that the research was conducted in the absence of any commercial or financial relationships that could be construed as a potential conflict of interest.

## Footnotes

^1^It may be an interesting task to catalog objective cues in a situation and contrast these with the cues participants noticed and reported. Of course, participants cannot process all cues and will not find all of them relevant (or recall them). Thus, the selection of cues that participants nominate are likely the most important and salient ones to them (e.g., a book), and these may be tied strongly to perceived situation characteristics (e.g., intellectual).

^2^In principle, a situation could also belong to (two or more classes). However, for simplicity sake and illustrative purposes, our example assumes that a situation can only belong to one class at a time.

^3^If situations are engendered in an intentional and/or goal-directed way, then they utilize modification or creation.

^4^By not doing anything, people can elicit adverse situations (see, e.g., laissez-faire leadership: Furtner et al., 2013). Thus, evocation does not require physical activity to take place. Should a person, however, actively “provoke” a situation, then this falls under either situation modification (if the provoked situation is an escalation of an already existing one) or situation creation (if the provoked situation is created out of the blue).
