# Supplementary material for: Situation Change: Stability and Change of Situation Variables between and within Persons
Source: Front Psychol. 2016 Jan 6;6:1938. doi: 10.3389/fpsyg.2015.01938 (PMC4703053; doi:10.3389/fpsyg.2015.01938)
Supplement: Supplementary file 1 [file Presentation1.zip › presentation/Figures/Figure 1.pptx]

## Slide 1
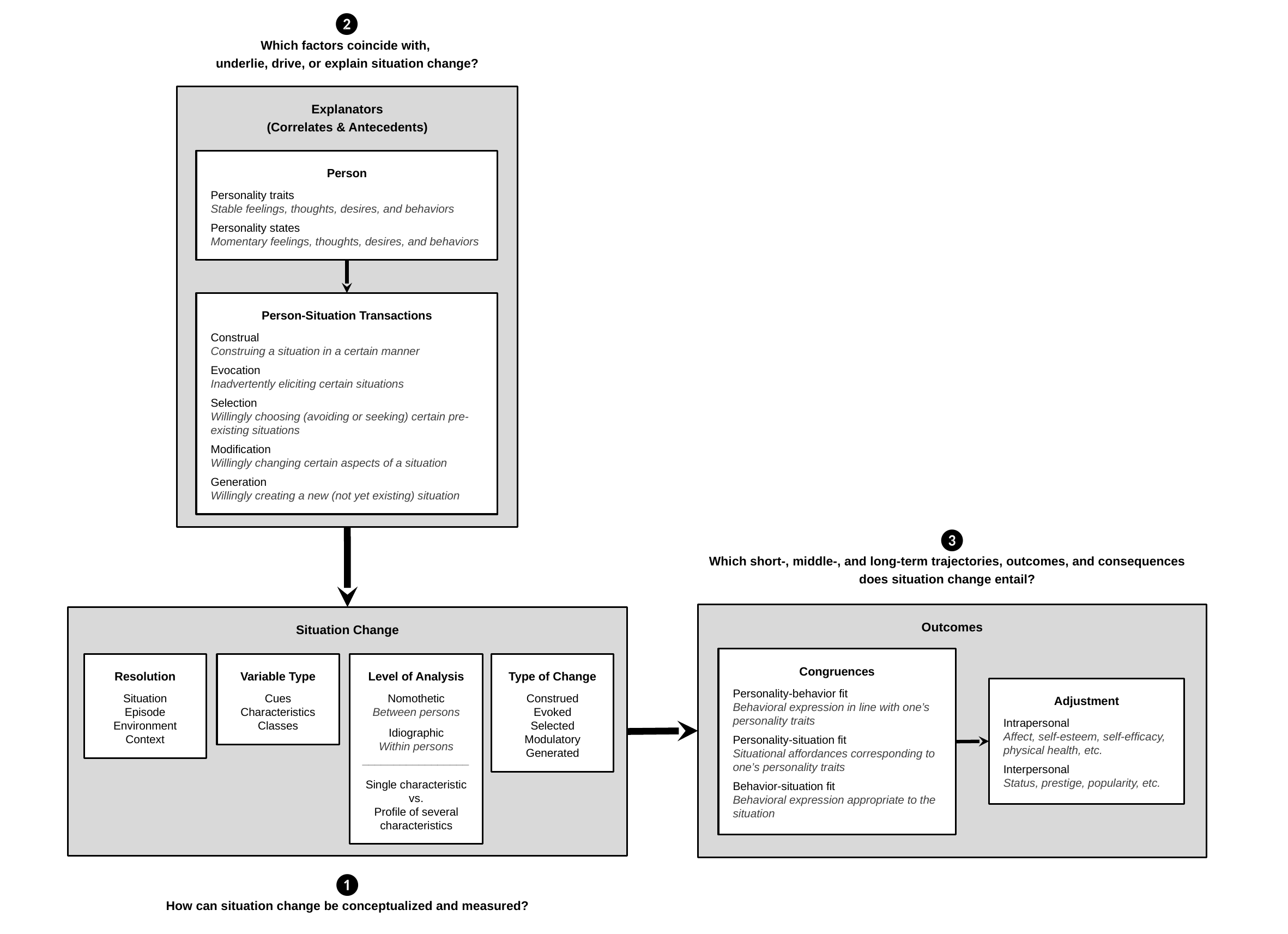

❷
Which factors coincide with,
underlie, drive, or explain situation change?
Explanators
(Correlates & Antecedents)
Person
Personality traits
Stable feelings, thoughts, desires, and behaviors
Personality states
Momentary feelings, thoughts, desires, and behaviors
Person-Situation Transactions
Construal
Construing a situation in a certain manner
Evocation
Inadvertently eliciting certain situations
Selection
Willingly choosing (avoiding or seeking) certain pre-existing situations
Modification
Willingly changing certain aspects of a situation
Generation
Willingly creating a new (not yet existing) situation
❸
Which short-, middle-, and long-term trajectories, outcomes, and consequences
does situation change entail?
Outcomes
Situation Change
Congruences
Personality-behavior fit
Behavioral expression in line with one’s personality traits
Personality-situation fit
Situational affordances corresponding to one’s personality traits
Behavior-situation fit
Behavioral expression appropriate to the situation
Resolution
Situation
Episode
Environment
Context
Variable Type
Cues
Characteristics
Classes
Level of Analysis
Nomothetic
Between persons
Idiographic
Within persons
_________________
Single characteristic
vs.
Profile of several characteristics
Type of Change
Construed
Evoked
Selected
Modulatory
Generated
Adjustment
Intrapersonal
Affect, self-esteem, self-efficacy, physical health, etc.
Interpersonal
Status, prestige, popularity, etc.
❶
How can situation change be conceptualized and measured?
