# Supplementary material for: Situation Change: Stability and Change of Situation Variables between and within Persons
Source: Front Psychol. 2016 Jan 6;6:1938. doi: 10.3389/fpsyg.2015.01938 (PMC4703053; doi:10.3389/fpsyg.2015.01938)
Supplement: Supplementary file 1 [file Presentation1.zip › presentation/Figures/Figure 2.pptx]

## Slide 1
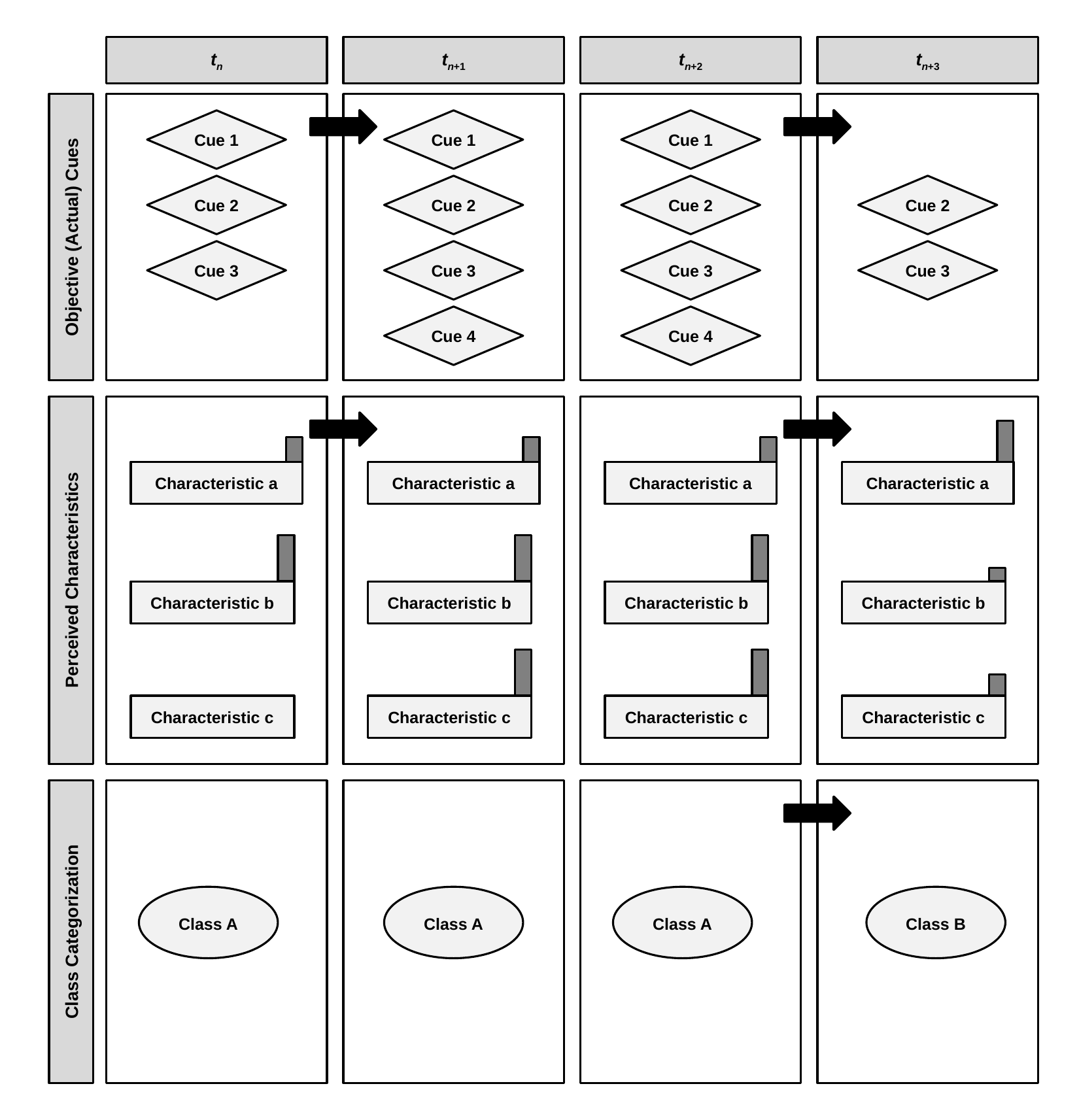

tn
tn+1
tn+2
tn+3
Cue 1
Cue 1
Cue 1
Cue 2
Cue 2
Cue 2
Cue 2
Objective (Actual) Cues
Cue 3
Cue 3
Cue 3
Cue 3
Cue 4
Cue 4
Characteristic a
Characteristic a
Characteristic a
Characteristic a
Perceived Characteristics
Characteristic b
Characteristic b
Characteristic b
Characteristic b
Characteristic c
Characteristic c
Characteristic c
Characteristic c
Class A
Class A
Class A
Class B
Class Categorization
