# Supplementary material for: Situation Change: Stability and Change of Situation Variables between and within Persons
Source: Front Psychol. 2016 Jan 6;6:1938. doi: 10.3389/fpsyg.2015.01938 (PMC4703053; doi:10.3389/fpsyg.2015.01938)
Supplement: Supplementary file 1 [file Presentation1.zip › presentation/Figures/Figure 3.pptx]

## Slide 1
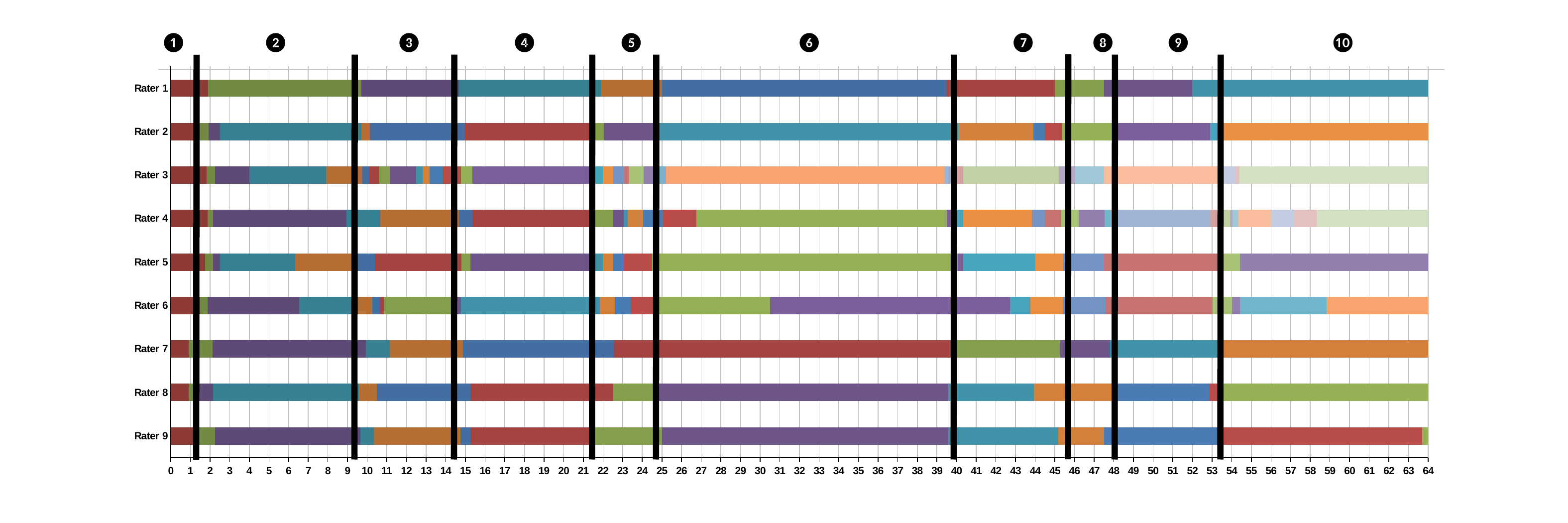

❶ ❷ ❸ ❹ ❺ ❻ ❼ ❽ ❾ ❿
### Chart
| Category | | | | | | | | | | | | | | | | | | | | | | | | | | | | | | | | | |
|---|---|---|---|---|---|---|---|---|---|---|---|---|---|---|---|---|---|---|---|---|---|---|---|---|---|---|---|---|---|---|---|---|---|
| Rater 9 | 0.0 | 1.1666666666666667 | 1.0833333333333333 | 7.416666666666666 | 0.6666666666666679 | 4.416666666666666 | 0.5 | 6.25 | 3.5 | 14.56666666666667 | 5.599999999999994 | 2.3333333333333357 | 5.766666666666666 | 10.433333333333337 | 0.29999999999999716 | None | None | None | None | None | None | None | None | None | None | None | None | None | None | None | None | None | None |
| Rater 8 | 0.0 | 0.9166666666666666 | 0.29999999999999993 | 0.95 | 7.416666666666668 | 0.9166666666666661 | 4.75 | 7.283333333333335 | 2.216666666666665 | 14.833333333333336 | 4.349999999999994 | 4.216666666666669 | 4.733333333333334 | 0.36666666666666714 | 10.75 | None | None | None | None | None | None | None | None | None | None | None | None | None | None | None | None | None | None |
| Rater 7 | 0.0 | 0.9166666666666666 | 1.2166666666666668 | 7.800000000000001 | 1.2166666666666668 | 3.7333333333333325 | 7.666666666666668 | 17.23333333333333 | 5.483333333333334 | 2.5500000000000043 | 5.633333333333333 | 10.549999999999997 | None | None | None | None | None | None | None | None | None | None | None | None | None | None | None | None | None | None | None | None | None |
| Rater 6 | 0.0 | 1.5 | 0.3833333333333333 | 4.666666666666666 | 2.7333333333333334 | 0.9666666666666668 | 0.38333333333333286 | 0.20000000000000107 | 3.4499999999999993 | 0.5 | 7.049999999999999 | 0.7666666666666693 | 0.7999999999999972 | 1.3500000000000014 | 5.75 | 12.216666666666669 | 1.0499999999999972 | 1.6333333333333329 | 2.183333333333337 | 5.449999999999996 | 0.9833333333333343 | 0.3999999999999986 | 4.416666666666671 | 5.166666666666664 | None | None | None | None | None | None | None | None | None |
| Rater 5 | 0.0 | 1.75 | 0.3999999999999999 | 0.3500000000000001 | 3.833333333333333 | 2.9666666666666677 | 1.0833333333333321 | 4.416666666666668 | 0.4499999999999993 | 6.25 | 0.5 | 0.533333333333335 | 0.5499999999999972 | 1.4166666666666679 | 15.56666666666667 | 0.2666666666666657 | 3.6666666666666643 | 1.4166666666666643 | 2.0833333333333357 | 5.950000000000003 | 0.9666666666666615 | 9.583333333333336 | None | None | None | None | None | None | None | None | None | None | None |
| Rater 4 | 0.0 | 1.8833333333333333 | 0.2833333333333332 | 6.783333333333333 | 1.7166666666666668 | 4.0 | 0.7000000000000011 | 6.133333333333333 | 1.033333333333335 | 0.5333333333333314 | 0.18333333333333357 | 0.8000000000000007 | 1.0 | 1.716666666666665 | 12.733333333333334 | 0.3333333333333357 | 0.5 | 3.5 | 0.6499999999999986 | 0.8500000000000014 | 0.8833333333333329 | 1.3166666666666629 | 0.30000000000000426 | 0.3333333333333286 | 4.75 | 0.4000000000000057 | 0.5999999999999943 | 0.13333333333333286 | 0.28333333333333854 | 1.6666666666666643 | 1.18333333333333 | 1.1500000000000057 | 5.666666666666664 |
| Rater 3 | 0.0 | 1.8333333333333333 | 0.41666666666666674 | 1.75 | 3.916666666666667 | 1.833333333333333 | 0.3333333333333339 | 0.5333333333333332 | 0.5499999999999989 | 1.333333333333334 | 0.3333333333333339 | 0.34999999999999964 | 0.6500000000000004 | 0.9499999999999993 | 0.5666666666666664 | 6.183333333333335 | 0.466666666666665 | 0.533333333333335 | 0.5499999999999972 | 0.23333333333333428 | 0.75 | 0.5833333333333321 | 0.5500000000000007 | 14.2 | 0.5500000000000043 | 0.38333333333333286 | 4.866666666666667 | 0.7999999999999972 | 1.5166666666666657 | 5.800000000000004 | 0.86666666666666 | 0.21666666666666856 | 9.600000000000001 |
| Rater 2 | 0.0 | 1.25 | 0.6833333333333333 | 0.5666666666666667 | 7.199999999999999 | 0.43333333333333357 | 4.833333333333334 | 6.616666666666665 | 0.45000000000000284 | 2.6499999999999986 | 15.400000000000002 | 3.816666666666663 | 0.6000000000000014 | 0.8833333333333329 | 2.56666666666667 | 4.949999999999996 | 0.4166666666666714 | 10.68333333333333 | None | None | None | None | None | None | None | None | None | None | None | None | None | None | None |
| Rater 1 | 0.0 | 1.9 | 7.799999999999999 | 4.966666666666667 | 7.2333333333333325 | 3.1000000000000014 | 14.5 | 5.5 | 2.5 | 4.5 | 12.0 | None | None | None | None | None | None | None | None | None | None | None | None | None | None | None | None | None | None | None | None | None | None |
