# Supplementary figures and images for: Situation Change: Stability and Change of Situation Variables between and within Persons
Source: Front Psychol. 2016 Jan 6;6:1938. doi: 10.3389/fpsyg.2015.01938 (PMC4703053; doi:10.3389/fpsyg.2015.01938)

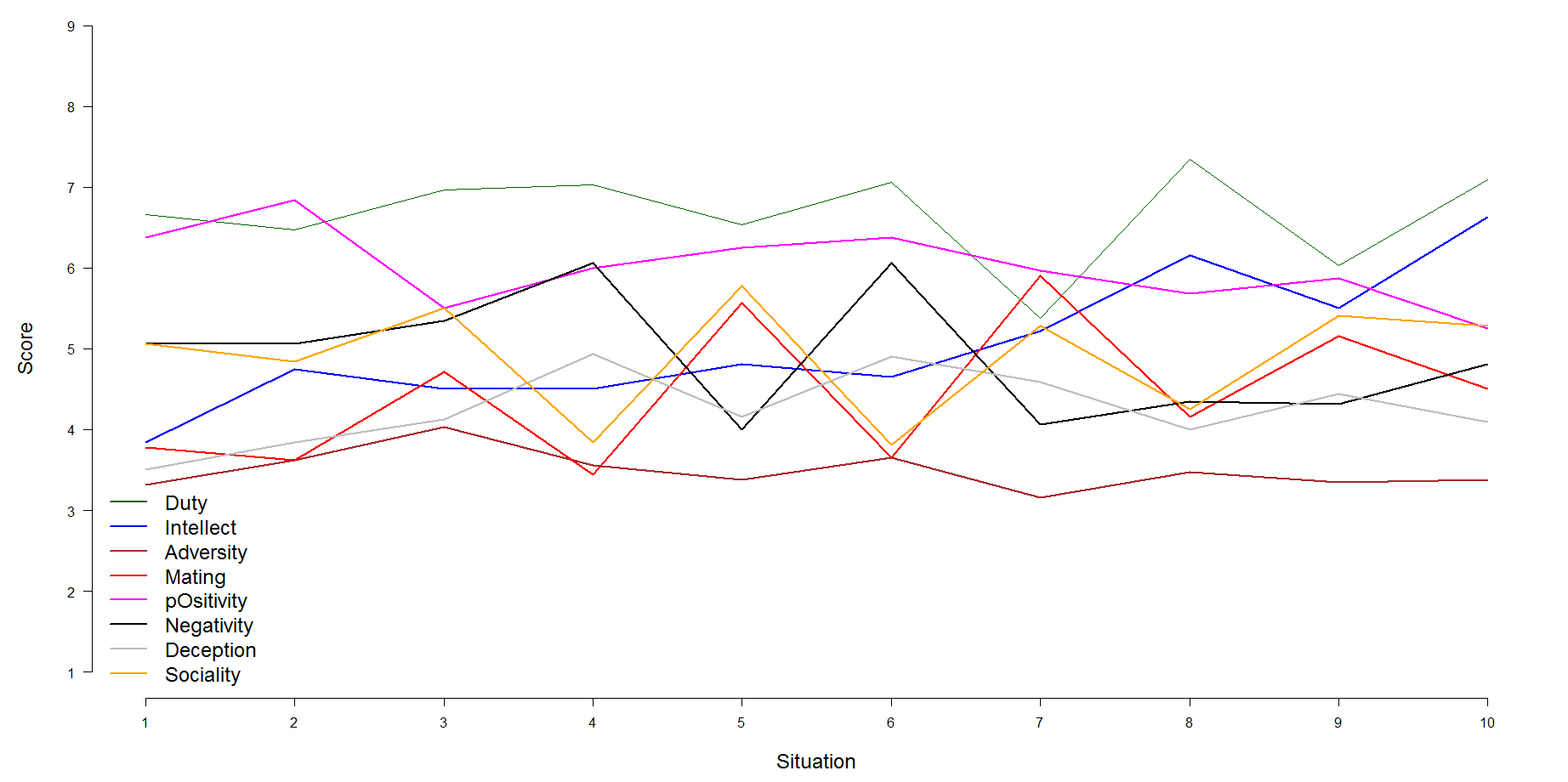

Supplement: Supplementary file 1 [file Presentation1.zip › presentation/Figures/Figure 4.tiff]

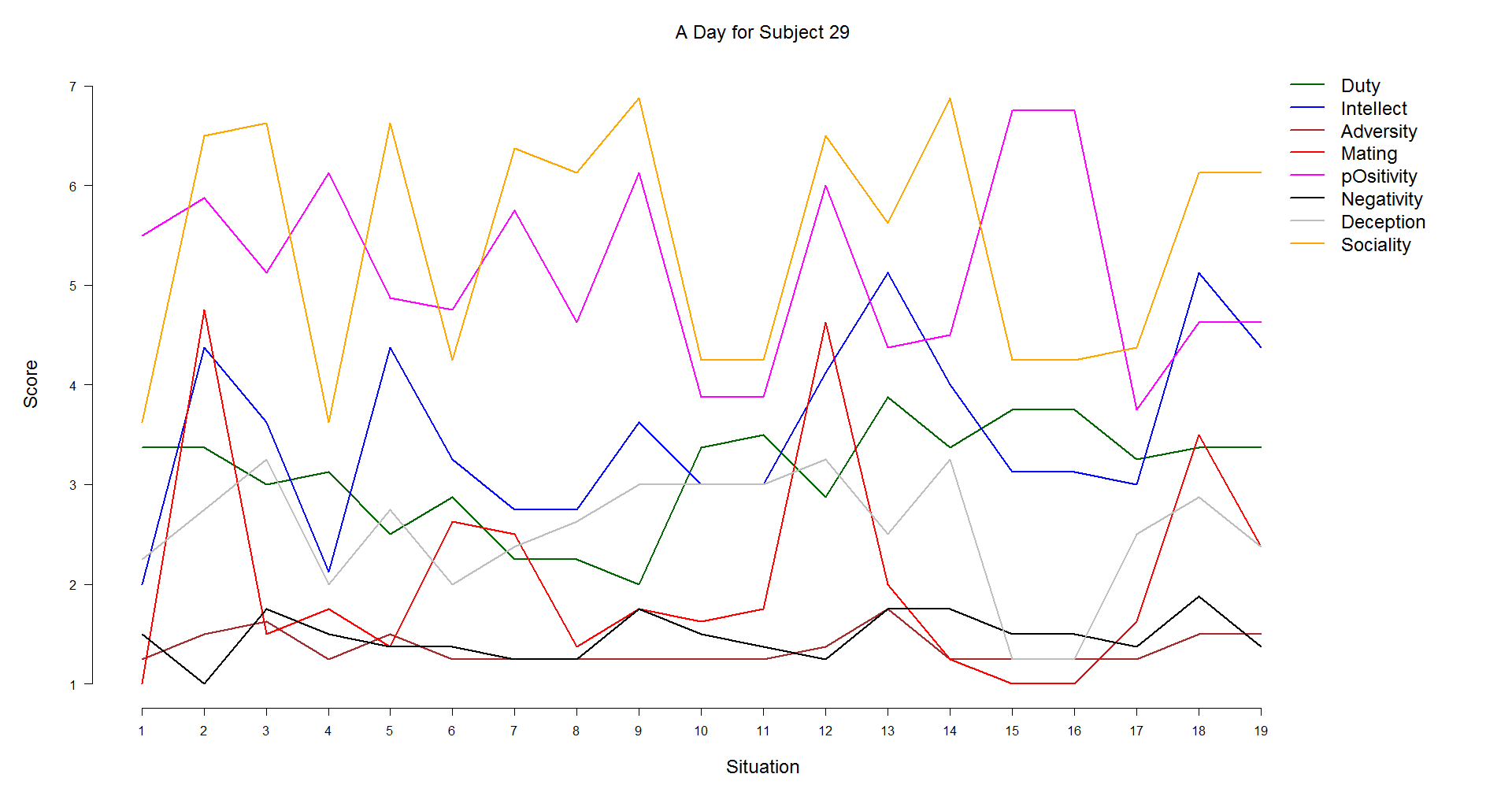

Supplement: Supplementary file 1 [file Presentation1.zip › presentation/Figures/Figure 5a.tiff]

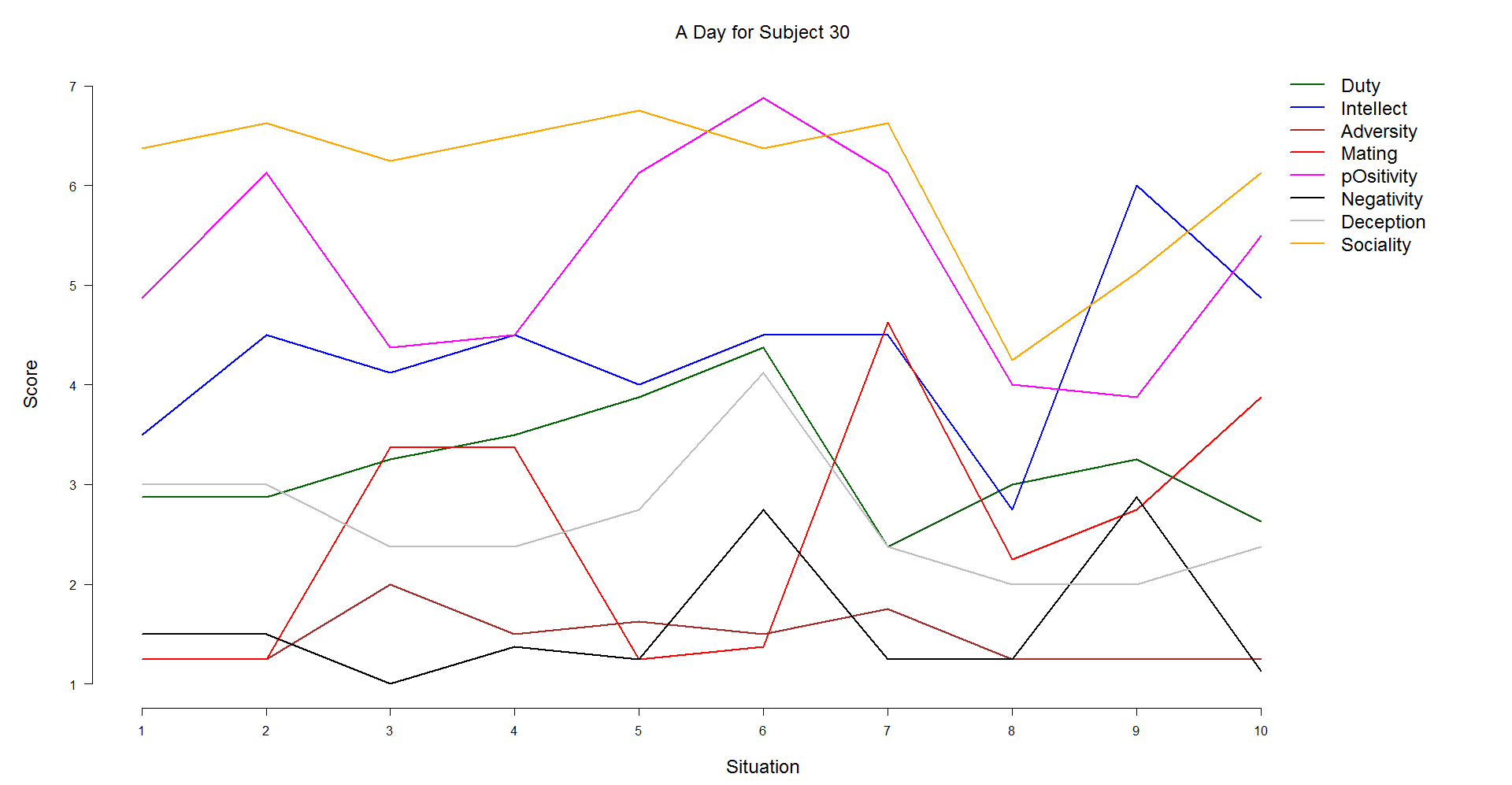

Supplement: Supplementary file 1 [file Presentation1.zip › presentation/Figures/Figure 5b.tiff]

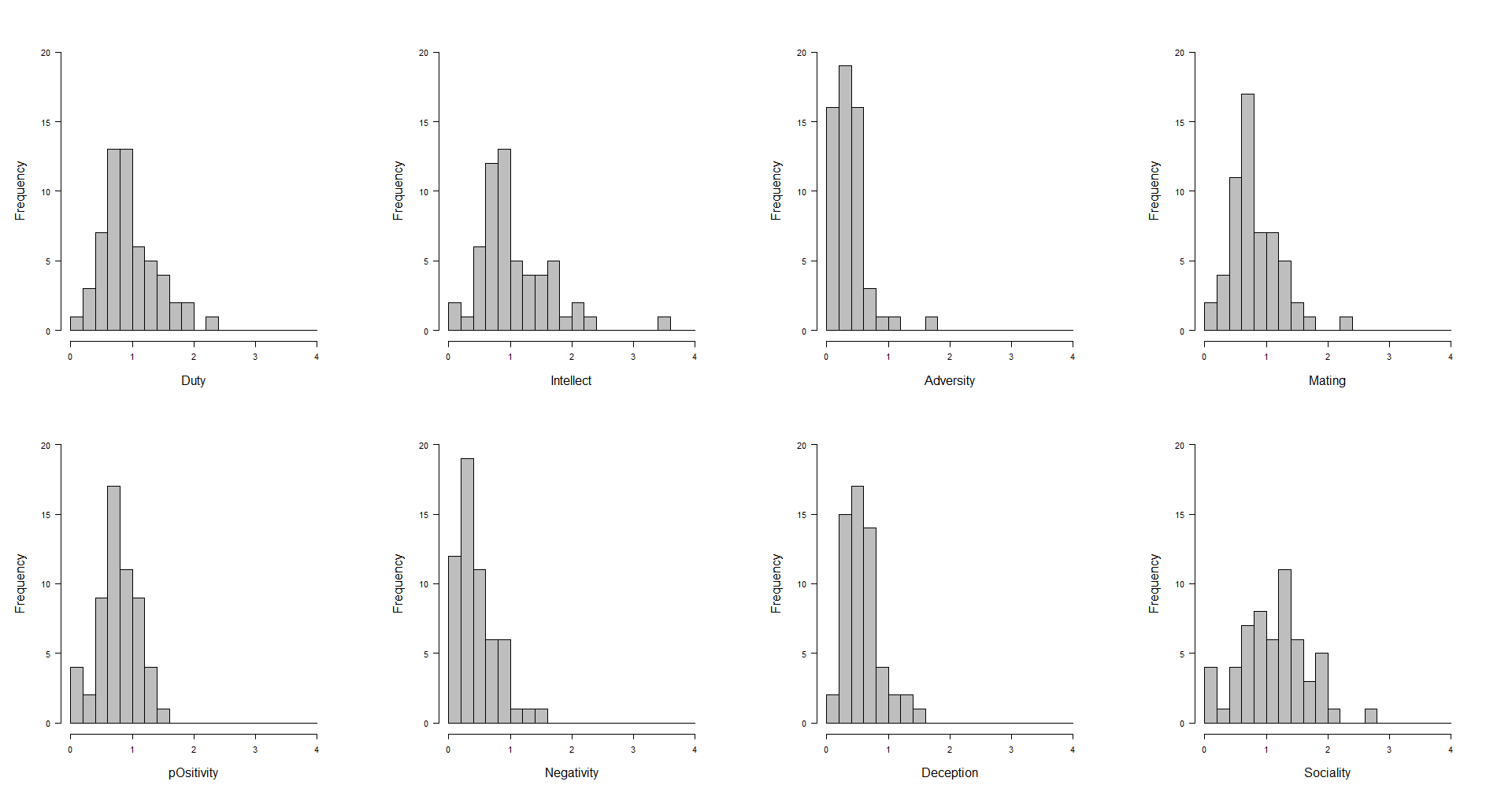

Supplement: Supplementary file 1 [file Presentation1.zip › presentation/Figures/Figure 6.tiff]

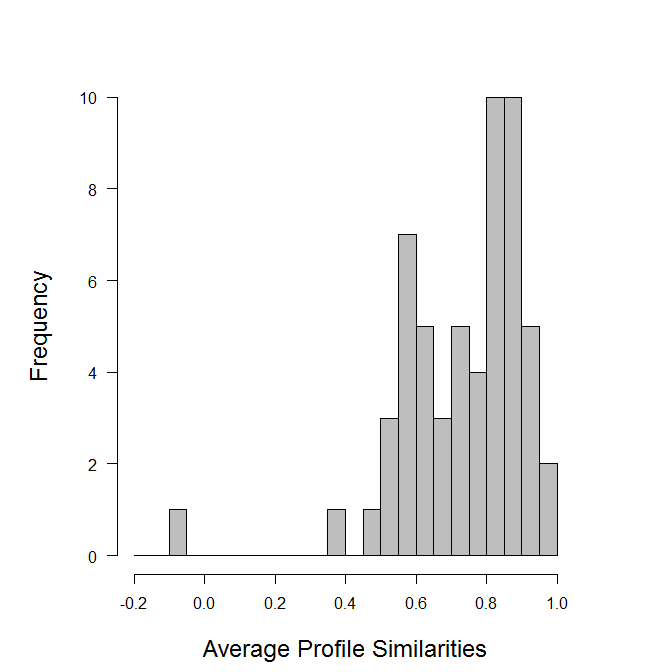

Supplement: Supplementary file 1 [file Presentation1.zip › presentation/Figures/Figure 7.tiff]

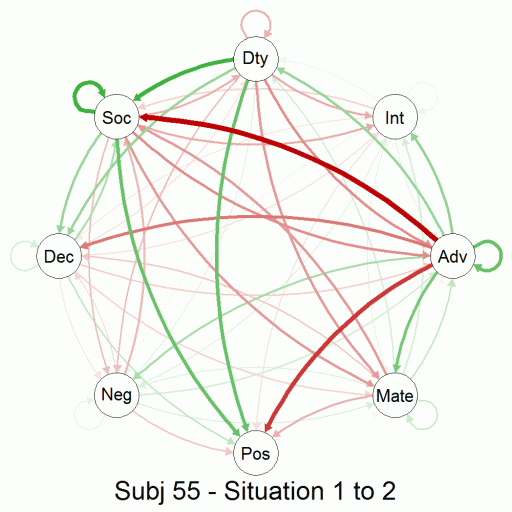

Supplement: Supplementary file 1 [file Presentation1.zip › presentation/Figures/Figure 8a.gif]

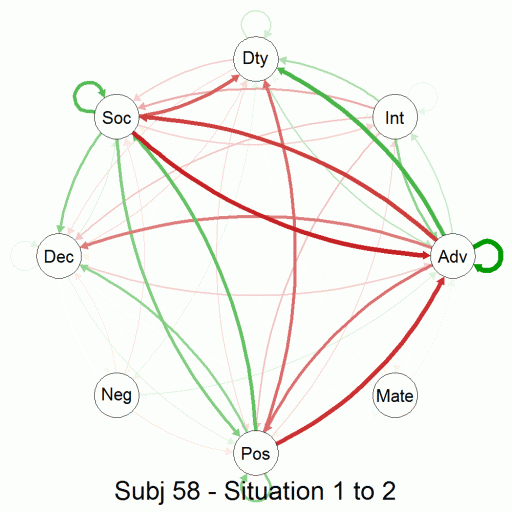

Supplement: Supplementary file 1 [file Presentation1.zip › presentation/Figures/Figure 8b.gif]

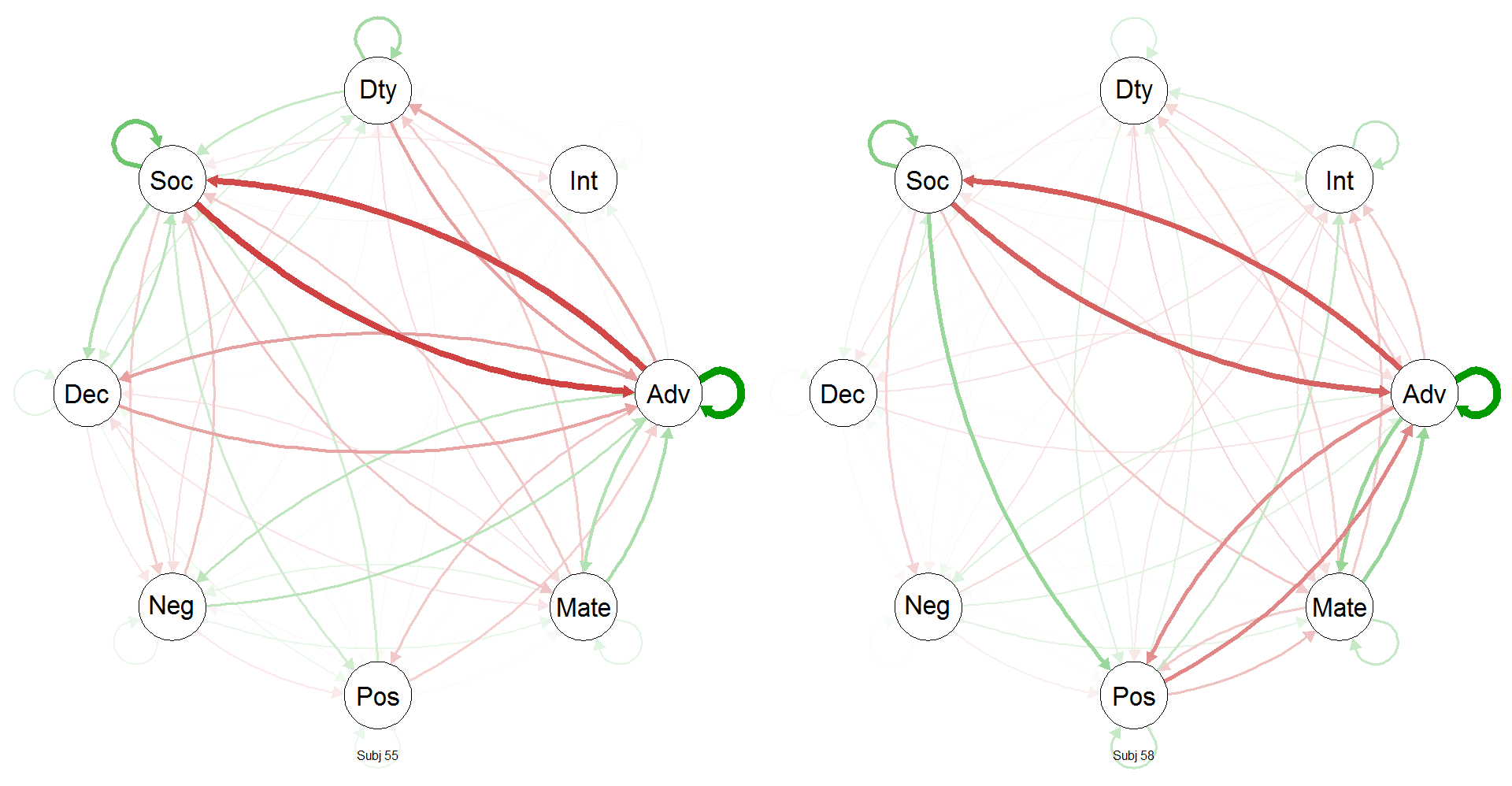

Supplement: Supplementary file 1 [file Presentation1.zip › presentation/Figures/Figure 9.tiff]
